# A microphysiological system reveals neutrophil contact-dependent attenuation of pancreatic tumor progression by CXCR2 inhibition-based immunotherapy

**DOI:** 10.1038/s41598-024-64780-4

**Published:** 2024-06-19

**Authors:** Shuai Shao, Nikki A. Delk, Caroline N. Jones

**Affiliations:** 1 Department of Bioengineering, The University of Texas at Dallas, Richardson, TX 75080 USA; 2 Department of Biomedical Engineering, UT Southwestern Medical Center, Dallas, TX 75235 USA; 3 Department of Biological Sciences, The University of Texas at Dallas, Richardson, TX 75080 USA

**Keywords:** Cancer, Immunology, Engineering

## Abstract

Cancer cells recruit neutrophils from the bloodstream into the tumor tissue, where these immune cells promote the progression of numerous solid tumors. Studies in mice suggest that blocking neutrophil recruitment to tumors by inhibition of neutrophil chemokine receptor CXCR2 could be a potential immunotherapy for pancreatic cancer. Yet, the mechanisms by which neutrophils promote tumor progression in humans, as well as how CXCR2 inhibition could potentially serve as a cancer therapy, remain elusive. In this study, we developed a human cell-based microphysiological system to quantify neutrophil-tumor spheroid interactions in both “separated” and “contact” scenarios. We found that neutrophils promote the invasion of tumor spheroids through the secretion of soluble factors and direct contact with cancer cells. However, they promote the proliferation of tumor spheroids solely through direct contact. Interestingly, treatment with AZD-5069, a CXCR2 inhibitor, attenuates invasion and proliferation of tumor spheroids by blocking direct contact with neutrophils. Our findings also show that CXCR2 inhibition reduces neutrophil migration toward tumor spheroids. These results shed new light on the tumor-promoting mechanisms of human neutrophils and the tumor-suppressive mechanisms of CXCR2 inhibition in pancreatic cancer and may aid in the design and optimization of novel immunotherapeutic strategies based on neutrophils.

## Introduction

Neutrophils are the most abundant immune cells in human circulation and the first line of defense against microbial infection^1–4^. Cancer cells can also recruit neutrophils from circulation into the tumor tissue and modulate them to promote tumor progression^1,5–12^. Neutrophils present in solid tumor tissues, known as tumor-associated neutrophils (TANs), have been reported to promote tumor proliferation, angiogenesis, and metastasis, thereby subverting the canonical role of neutrophils to protect the body from harm^1,5–12^. One of these solid tumors is pancreatic ductal adenocarcinoma (PDAC), the most common type of pancreatic cancer with an extremely poor prognosis reaching a 5-year survival rate of only 9%^1,6–8,13^. Both high neutrophil-to-lymphocyte ratio in peripheral blood and high neutrophil infiltration in tumor correlate with poor prognosis in PDAC patients^14^ and with worse responses to chemotherapy, radiotherapy, and T cell-based immunotherapy in various human cancer types^1,2^. Neutrophils have been recently explored as a novel promising therapeutic target for cancer in different ways, one of them being blocking the recruitment of neutrophils to tumor sites^1^. Particularly, inhibition of C-X-C Motif Chemokine Receptor 2 (CXCR2), a neutrophil chemokine receptor, was shown to reduce neutrophil recruitment to tumor, increase T cell infiltration into the tumor, and improve the responses to chemotherapy and T cell-based immunotherapy in pancreatic cancer-bearing mice^15–21^. Based on encouraging results from preclinical mouse studies, four CXCR2 inhibitors have now entered clinical trials for treatment of cancer^22^. One clinical trial in particular aims to evaluate nab-paclitaxel and gemcitabine chemotherapy in combination with AZD-5069, a small-molecule CXCR2 antagonist, in metastatic pancreatic cancer (NCT02583477). Hence, blockade of neutrophil recruitment via CXCR2 inhibition seems to be one of the most promising neutrophil-targeting immunotherapies^1–3^.

However, both the mechanisms by which neutrophils play a tumor-promoting role in humans and the mechanisms by which CXCR2 inhibition might have a tumor-suppressive effect in human cancer are not completely understood^1,10^. Although neutrophils have been found to play a tumor-promoting role through release of a variety of soluble factors^23^, it is unclear whether neutrophils could promote tumor progression also through direct contact with cancer cells. Although CXCR2 inhibition was shown to reduce neutrophil recruitment to tumor in mice^15–21^ and in in vitro models of human lung cancer and breast cancer^24,25^, it is unknown whether CXCR2 inhibition could also reduce neutrophil recruitment to human pancreatic tumor tissue. An important question is whether CXCR2 inhibition attenuates neutrophil-promoted tumor progression via blocking release of soluble factors by neutrophils or blocking direct contact between neutrophils and cancer cells. Indeed, soluble factors have been the major focus of inflammation research and the targets of therapeutic interventions for a long time^26^. As a second mechanism of intercellular communication, direct cell–cell contact is also an essential mediator in inflammation and a potential therapeutic target for inflammatory diseases including cancer^26^. However, the tools and techniques for studying direct cell–cell contact are far more limited than those for studying soluble factors ranging from enzyme-linked immunosorbent assays (ELISA) to chromatography^26^. The intrinsic complexity of animal models makes it difficult to isolate and analyze the roles of specific cell–cell interactions within the tumor microenvironment (TME), including those between neutrophils and cancer cells^27^. Furthermore, understanding specific types of interactions, such as the release of soluble factors versus direct cell–cell contact, in tumor progression in vivo also presents challenges^27^. Traditional in vitro models used for examining cell–cell interactions, like those using conditioned media, fail to enable mutual communication between different cell types. On the other hand, methods such as bulk co-cultures lack spatial control and compartmentalization, while approaches like transwell assays provide only general measurements without single-cell resolution and only offer end-point data without insights into the temporal dynamics of cellular behaviors^28–30^. Therefore, the limitations of currently available traditional tools render a full depiction of the tumor-promoting mechanisms of neutrophils and the tumor-suppressive mechanisms of CXCR2 inhibition challenging.

**Figure 1:**
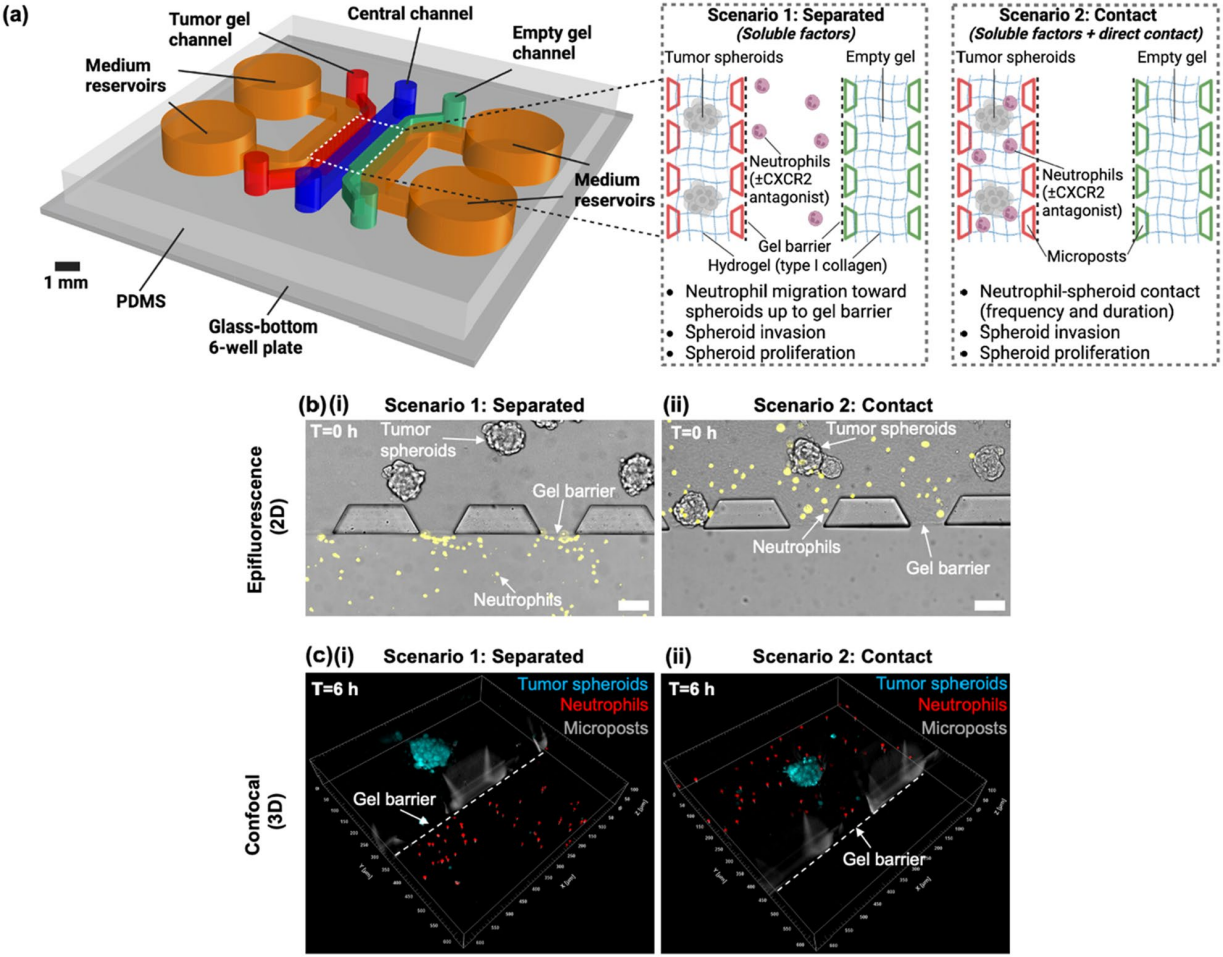
Design, characterization, and validation of the “neutrophil-tumor interactions on-a-chip” (NTI-chip) microphysiological system. (**a**) The NTI-chip models neutrophil interactions with 3D tumor spheroids embedded in collagen hydrogel in two independent scenarios, “separated” and “contact”, to tease out release of soluble factors and direct cell–cell contact as two different tumor-promoting mechanisms of neutrophils. The parameters measured in each scenario are listed below the schematic. Created with Rhino 7 and BioRender.com. (**b**) Representative 10X epifluorescence images of tumor spheroids (unstained) and neutrophils (yellow, CMRA) in “separated” (**i**) and “contact” (**ii**) scenarios at t = 0 h. Scale bar, 100 µm. (**c**) 3D rendering of representative 20X confocal images of tumor spheroids (blue, CMAC) and neutrophils (red, CMRA) in “separated” (**i**) and “contact” (**ii**) scenarios fixed at t = 6 h. Z-stacks were taken with a 2 μm step size. X-y-z coordinates are shown around each field of view.

Tissue-engineered microfluidics-based systems, also known as microphysiological systems, have emerged as novel in vitro models to overcome the limitations of traditional models^7,29,30^. These systems can be designed to integrate the features of physiological relevance, single-cell resolution, temporal dynamics, and precise spatial control within one assay^7,29,30^. As a result, microphysiological systems have been utilized to investigate the roles of soluble factors and direct contact in cell–cell interactions associated with tumor progression (Supplementary Table S1)^31–35^. Microphysiological systems have also been utilized to investigate the role of neutrophils in various processes associated with tumor progression (Supplementary Table S1)^25,36–42^. However, none of these previous studies examined the role of direct contact in neutrophil promotion of pancreatic tumor progression. In this study, we engineered a human cell-based, reductionist microphysiological system “neutrophil-tumor interactions on-a-chip” (NTI-chip) to monitor in real-time and measure interactions between neutrophils and 3D hydrogel-embedded pancreatic tumor spheroids (Fig. 1a). We constructed both “separated” (where neutrophils were physically separated from tumor spheroids) and “contact” (where neutrophils were allowed to engage in direct contact with tumor spheroids) scenarios to spatially control the contact between neutrophils and tumor spheroids. This engineering design helped reveal the important role of direct contact in the tumor-promoting mechanisms of neutrophils and the tumor-suppressive mechanisms of CXCR2 inhibition. The NTI-chip enabled us to quantify neutrophil migration toward tumor spheroids, neutrophil contact with tumor spheroids, and tumor spheroid progression in terms of invasion and proliferation (Fig. 1a). The NTI-chip provides valuable temporal information of neutrophil-tumor interactions that would be lacking in standard end-point molecular biology assays and may serve as a complementary tool to animal models for preclinical testing of novel neutrophil-based immunotherapies.

## Results

### Design, characterization, and validation of the NTI-chip

To dissect the tumor-promoting mechanisms of neutrophils and the tumor-suppressive mechanisms of CXCR2 inhibition, we developed a microphysiological system “NTI-chip” with two independent scenarios, “separated” and “contact”. The NTI-chip comprised a central channel (*blue*), a tumor gel channel (*red*), an empty gel channel (*green*), and two outermost medium channels, each having two medium reservoirs for nutrient supply and to prevent evaporation (*orange*) (Fig. 1a). The five parallel channels were compartmentalized and interconnected by regularly spaced trapezoidal microposts, allowing diffusion of soluble factors between channels (Fig. 1a). In the “separated” scenario, the central channel housed neutrophils in culture medium and the tumor gel channel housed tumor spheroids embedded in a 3D hydrogel which mimics the extracellular matrix (ECM) of the in vivo tumor tissue (Fig. 1a,bi,ci). The liquid-gel interface between the two channels acted as a physical barrier that prevented neutrophils from migrating into the gel channel and thus helped maintain the “separated” nature of the scenario^43,44^. We demonstrated that 10 kDa fluorescein-dextran can diffuse from the central channel into the tumor gel channel within 1 h and from the tumor gel channel into the central channel within 1 h (Supplementary Fig. S1). Hence, we inferred that soluble factors released by tumor spheroids in the tumor gel channel can reach neutrophils in the central channel and that soluble factors released by neutrophils in the central channel can reach tumor spheroids in the tumor gel channel. With this setup, neutrophils and tumor spheroids were physically separated from each other but still allowed to communicate through release of soluble factors. In the “contact” scenario, the tumor gel channel housed both neutrophils and tumor spheroids embedded in the same 3D hydrogel, allowing the two cell types to communicate via direct cell–cell contact in addition to release of soluble factors (Fig. 1a,bii,cii). The empty gel channel (*green*) housed empty hydrogel in both scenarios, acting as a negative control (Fig. 1a). We also characterized the spatiotemporal distribution and localization of neutrophils in the central channel in the “separated” scenario and those in the tumor gel channel in the “contact” scenario over 24 h (Supplementary Fig. S2). The precise spatial control of different cell types in this engineering design enabled us to tease out release of soluble factors and direct cell–cell contact as two different tumor-promoting mechanisms of neutrophils. By adding a CXCR2 antagonist AZD-5069 in both scenarios, the NTI-chip also enabled us to tease out blockade of soluble factors and blockade of direct cell–cell contact as two different tumor-suppressive mechanisms of CXCR2 inhibition.

**Figure 2:**
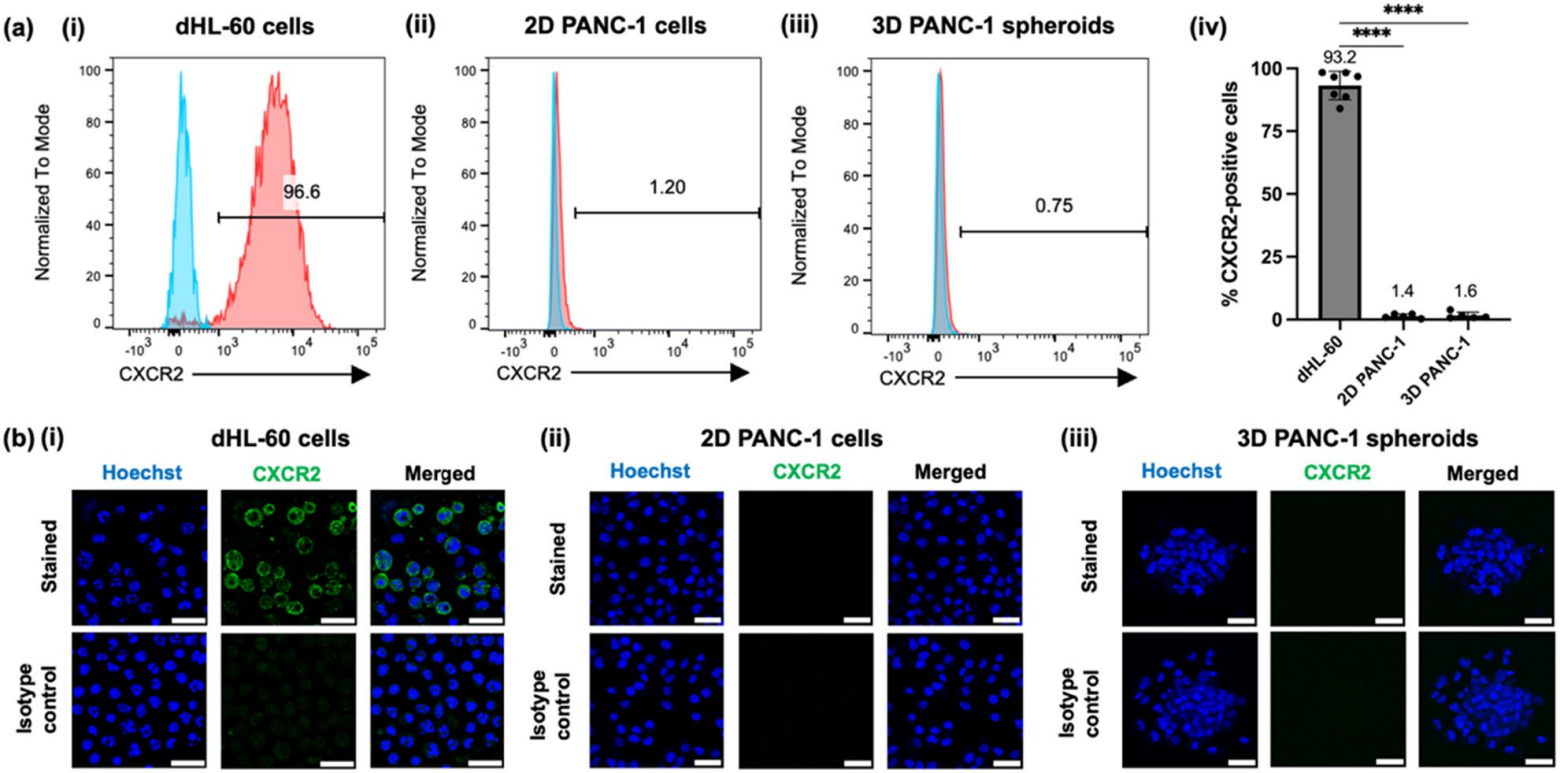
CXCR2 is expressed on the surface of dHL-60 cells but not PANC-1 cells. (**a**) ﻿CXCR2 expressions by dHL-60 cells (**i**), 2D PANC-1 cells (**ii**), and dissociated 3D PANC-1 spheroids (**iii**) were measured by flow cytometry. Red histogram = stained sample; blue histogram = unstained control. The horizontal bars show percentages of CXCR2-positive cells. One representative experiment is shown. (**iv**) Bar plot showing the percentage of CXCR2-positive cells in dHL-60 cells, 2D PANC-1 cells, and 3D PANC-1 spheroids from at least three independent experiments. Bars show mean ± SD with mean values written above. ****: *p* < 0.0001, Brown-Forsythe and Welch ANOVA with Dunnett’s T3 multiple comparisons test. (**b**) CXCR2 expressions by dHL-60 cells (**i**), 2D PANC-1 cells (**ii**), and 3D PANC-1 spheroids (**iii**) were visualized by immunofluorescence. (**i**) Representative 40X confocal images of dHL-60 cells (blue, Hoechst) immunostained for CXCR2 (green). Scale bar, 20 µm. (**ii**) Representative 20X confocal images of 2D PANC-1 cells (blue, Hoechst) immunostained for CXCR2 (green). Scale bar, 50 µm. (**iii**) Representative 20X confocal images of gel-embedded 3D PANC-1 spheroids (blue, Hoechst) immunostained for CXCR2 (green). Images are maximum intensity projections of z-stacks with a 2 μm step size. Scale bar, 50 µm. Samples stained with the secondary antibody only were used as isotype control.

In this proof-of-concept study of neutrophil-tumor interactions, we used dimethyl sulfoxide (DMSO)-differentiated HL-60 cells (dHL-60 cells), a human ﻿promyelocytic leukemia cell line that has been extensively characterized and is the most commonly used model for primary human neutrophils^45,46^. Validation of CD11b expression by dHL-60 cells is described in Supplementary Results (Supplementary Fig. S3). We also confirmed dHL-60 cell expression of CXCR2, the main neutrophil chemokine receptor and the putative target of AZD-5069, using both flow cytometry (% CXCR2-positive cells = 93.2 ± 5.7) and immunofluorescence (Fig. 2ai,aiv,bi). We grew tumor spheroids from the PANC-1 human pancreatic cancer cell line on an ultra-low attachment (ULA) plate. The tumor spheroids were then embedded in biomimetic hydrogel made of type I collagen, which is the major component of the pancreatic cancer-specific ECM^47–49^ and is known to promote an invasive phenotype of pancreatic cancer cells^47,49^. Characterization and validation of tumor spheroid phenotypes in the NTI-chip are described in Supplementary Results (Supplementary Fig. S4). CXCR2 expression was hardly detected on the surface of PANC-1 cells in either 2D monolayer culture (% CXCR2-positive cells = 1.4 ± 0.8) (Fig. 2aii,aiv,bii) or 3D spheroid culture (% CXCR2-positive cells = 1.6 ± 1.3) (Fig. 2aiii,aiv,biii), based on both flow cytometry and immunofluorescence.

### dHL-60 cells show directional migration toward PANC-1 tumor spheroids in the “separated” scenario

**Figure 3:**
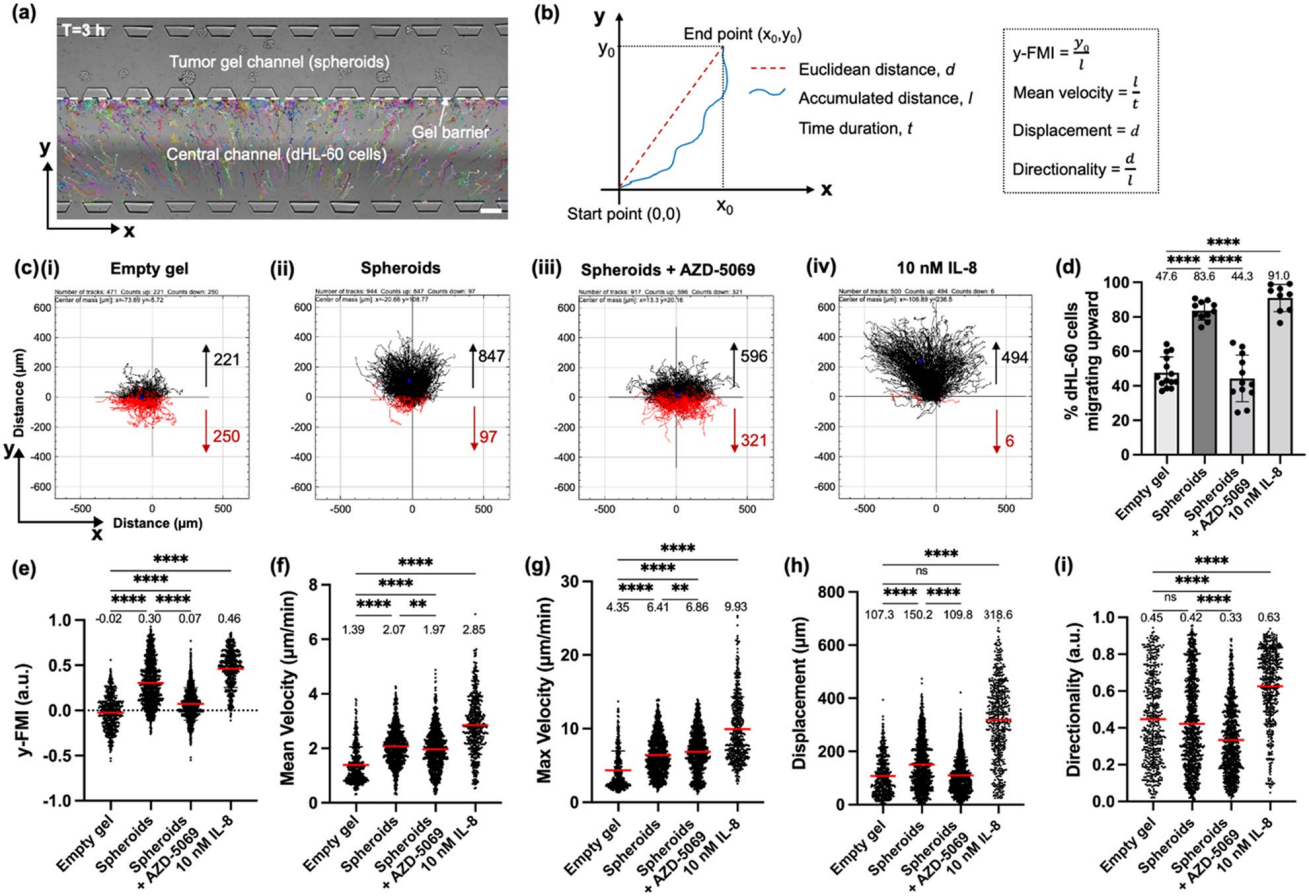
CXCR2 inhibition reduces dHL-60 cell migration toward PANC-1 tumor spheroids in the “separated” scenario. (**a**) A representative image showing dHL-60 cells (pseudocolor-coded) in the central channel migrating toward PANC-1 tumor spheroids (brightfield) in the tumor gel channel up to the gel barrier, overlaid with single-cell trajectories over 3 h extracted by TrackMate (ImageJ). Scale bar, 200 µm. The empty gel channel was always loaded with empty gel as a negative control in all conditions and thus not shown for simplicity. (**b**) An illustration of the calculation of y-FMI, mean velocity, displacement, and directionality of a single-cell trajectory. All trajectories are transformed by setting the start points to (0,0). (**c**) Representative trajectory plots of migrating dHL-60 cells treated with DMSO vehicle control (**i, ii, iv**) or 1 μM AZD-5069 (**iii**) in response to empty gel control (**i**), tumor spheroids (**ii, iii**), or 10 nM IL-8 (**iv**) in the tumor gel channel over 3 h. Numbers in black and in red represent numbers of dHL-60 cells migrating toward (upward) and away from (downward) different conditions, respectively. (**d**) Bar plot showing the percentage of dHL-60 cells migrating upward toward specified conditions per NTI-chip. N = 9–14 NTI-chips per condition. (**e-i**) Scatter plots depicting the y-FMI (**e**), mean velocity (**f**), maximum velocity (**g**), displacement (**h**)**,** and directionality (**i**) of migrating dHL-60 cells in specified conditions. Each data point represents a single cell and n = 471–944 cells tracked per condition. Bars show mean (red) ± SD (black) with mean values written above the points. At least three independent experiments were performed and results from one representative experiment are shown. ns: ≥ 0.05, **: *p* < 0.01, ****: *p* < 0.0001, Brown-Forsythe and Welch ANOVA with Games-Howell's multiple comparisons test.

We quantified the ability of PANC-1 tumor spheroids to induce dHL-60 cell migration in the “separated” scenario in the NTI-chip. First, we showed that there was no spontaneous fluid flow detected in the NTI-chip using 10-µm fluorescent particles, thus eliminating the possibility that the observed dHL-60 cell migration could be biased by any unintentional fluid flow (Supplementary Fig. S5). Then, we used 10 kDa fluorescein-dextran to show the presence of a concentration gradient across the central channel in the first 6 h. Thus, we inferred that chemoattractants released by tumor spheroids would form a gradient across the central channel and enable chemotaxis of dHL-60 cells toward tumor spheroids (Supplementary Fig. S1b). Human interleukin-8 (IL-8) was used as a positive control for the migration experiments since IL-8 is a CXCR2 ligand known to induce neutrophil chemotaxis^28,50^ and the main chemokine released by the tumor tissue to recruit neutrophils in vivo^11,12^. The migratory dynamics of dHL-60 cells in the central channel in response to gel-embedded tumor spheroids, empty gel control, or IL-8 positive control in the tumor gel channel were captured by time-lapse imaging and analyzed with TrackMate (ImageJ) to generate single-cell trajectories (Fig. 3a,c). We calculated the percentage of dHL-60 cells that migrated toward tumor spheroids or IL-8 per chip. To further probe the migratory behaviors of dHL-60 cells with single-cell resolution, we quantified the following migratory parameters of individual cell trajectories: forward migration index in the y-direction (y-FMI), mean velocity, maximum velocity, displacement, and directionality (definitions listed in Table 1 and illustrated in Fig. 3b).

**Table 1:** Definitions of single-cell migratory parameters.

Parameter	Definition	Significance	Unit	Value range
y-FMI	Forward migration index in the y-direction. The y-coordinate of the ending position of a given trajectory after setting the starting position as (0,0) divided by the accumulated distance	Efficiency of cell migration or attraction toward the chemoattractant source	Unitless	[− 1,1]
Mean velocity	Accumulated distance divided by time duration of a given trajectory	Overall speed of cell migration	µm/min	(0, ∞)
Maximum velocity	The highest instantaneous velocity during a given trajectory	Highest speed of cell migration	µm/min	(0, ∞)
Displacement	The length of the straight line between the starting and the ending positions of a given trajectory; Euclidean distance	Effective distance of cell migration	µm	[0, ∞)
Directionality	The ratio of the Euclidean distance and the accumulated distance between the starting and the ending positions of a given trajectory	Straightness or directional persistence of cell migration. Irrelevant of the location of the chemoattractant source	Unitless	[0,1]

We first observed that dHL-60 cell migration in response to PANC-1 tumor spheroids switched from chemotaxis during 0–3 h of time-lapse imaging to chemokinesis during 3–6 h, which was evidenced by a decrease in y-FMI (0.15 vs. 0.04), an increase in mean velocity (2.86 vs. 3.73 µm/min), a decrease in directionality (0.33 vs. 0.23), and a decrease in displacement (153.9 vs. 129.7 µm) (*p* < 0.0001)^51^ (Supplementary Fig. S6). This switch in migration modes suggests that the continual diffusion of chemoattractants secreted by tumor spheroids into the central channel and the empty gel channel caused the dwindling of the chemoattractant gradient across the NTI-chip over time (Supplementary Fig. S6f). As a result, dHL-60 cells may have gradually lost the ability of chemotaxis and switched to chemokinesis which occurs when cells sense an even chemoattractant concentration around themselves^51^. Thus, we analyzed and compared migratory parameters between different conditions in the first 3 h to capture only chemotaxis and exclude chemokinesis. We then found that a higher percentage of dHL-60 cells migrated toward tumor spheroids (83.6%) and IL-8 (90.0%) than toward empty gel (47.0%) (*p* < 0.0001) (Fig. 3ci,cii,civ; Fig. 3d). We found that tumor spheroids significantly increased the y-FMI (0.30 vs. − 0.02), mean velocity (2.07 vs. 1.39 µm/min), maximum velocity (6.41 vs. 4.35 µm/min), and displacement (150.2 vs. 107.3 µm) of dHL-60 cell migration compared to empty gel control (*p* < 0.0001) (Fig. 3e–h). Hence, PANC-1 tumor spheroids can induce robust chemotaxis of dHL-60 cells up to the gel barrier in the “separated” scenario in the NTI-chip.

### CXCR2 inhibition reduces dHL-60 cell migration toward PANC-1 tumor spheroids in the “separated” scenario

We then assessed the effect of AZD-5069 on dHL-60 cell migration toward PANC-1 tumor spheroids. We first confirmed that AZD-5069 (1 µM) alone does not impact dHL-60 cell migration in the absence of tumor spheroids in a transwell assay (Supplementary Fig. S7). Similarly, we calculated the percentage of dHL-60 cells that migrated toward tumor spheroids per chip in the absence or presence of AZD-5069 (1 µM) (Fig. 3d). We found that a lower percentage of dHL-60 cells migrated toward tumor spheroids when dHL-60 cells were treated with AZD-5069 (44.3%) than with DMSO vehicle control (83.6%) (*p* < 0.0001) (Fig. 3cii,ciii,d). For parameters with single-cell resolution, we found that AZD-5069 significantly reduced the y-FMI (0.07 vs. 0.30), mean velocity (1.97 vs. 2.07 µm/min, *p* < 0.01), displacement (109.8 vs. 150.2 µm), and directionality (0.33 vs. 0.42) of dHL-60 cell migration toward tumor spheroids (p < 0.0001) (Fig. 3e,f,h,i). Interestingly, except the displacement (109.8 µm vs. 107.3 µm, *p* = 0.9288), treatment with AZD-5069 did not fully restore dHL-60 cell migration parameters to the basal level in the empty gel condition in terms of the y-FMI (0.07 vs. -0.02), mean velocity (1.97 vs. 1.39 µm/min), maximum velocity (6.86 vs. 4.35 µm/min), and directionality (0.33 vs. 0.45) (*p* < 0.0001) (Fig. 3e–i). This result reflects the complexity of signals secreted by tumor spheroids compared to CXCR2 ligands only. It also suggests there may be other molecular mechanisms responsible for neutrophil recruitment to tumor spheroids besides the CXCR2 axis. This warrants further investigation and therapeutic treatments may require a cocktail of inhibitors for full blockade of neutrophil recruitment. Taken together, these results show that CXCR2 inhibition via AZD-5069 (1 µM) effectively reduces dHL-60 cell migration toward PANC-1 tumor spheroids in the NTI-chip. This corroborates the finding of previous in vivo studies that CXCR2 inhibition can reduce neutrophil mobilization and recruitment to pancreatic tumor tissue in mouse models^15–17^. This human cell-based finding shows clinical promise of AZD-5069 to block the recruitment of neutrophils to the vicinity of the tumor tissue in pancreatic cancer patients.

### dHL-60 cells promote invasion of PANC-1 tumor spheroids via both release of soluble factors and direct cell–cell contact

After finding that PANC-1 tumor spheroids elicited strong migratory responses from dHL-60 cells, we sought to examine whether dHL-60 cells could elicit any reciprocal response from PANC-1 tumor spheroids. Neutrophils have been found to promote tumor metastasis in pancreatic cancer in mice^14^ and promote invasion of human pancreatic cancer cells in transwell assays^52^. Tumor invasion into the stroma is a critical step in metastasis^53^, as cancer cells in the primary tumor site must first invade into the surrounding ECM before entering the bloodstream and finally seeding at a secondary site in a distant organ to complete metastasis^54^. Therefore, we utilized the NTI-chip to model tumor invasion as an early-stage behavior of metastasis under the influence of neutrophils. Specifically, we measured the physical invasion of PANC-1 tumor spheroids into the surrounding gel matrix in the absence or presence of dHL-60 cells in both “separated” and “contact” scenarios. Since dHL-60 cells could affect tumor spheroid invasion via release of soluble factors in both scenarios but via direct cell–cell contact only in the “contact” scenario, any difference in tumor spheroid invasion observed between the two scenarios would be attributed to direct cell–cell contact.

**Figure 4:**
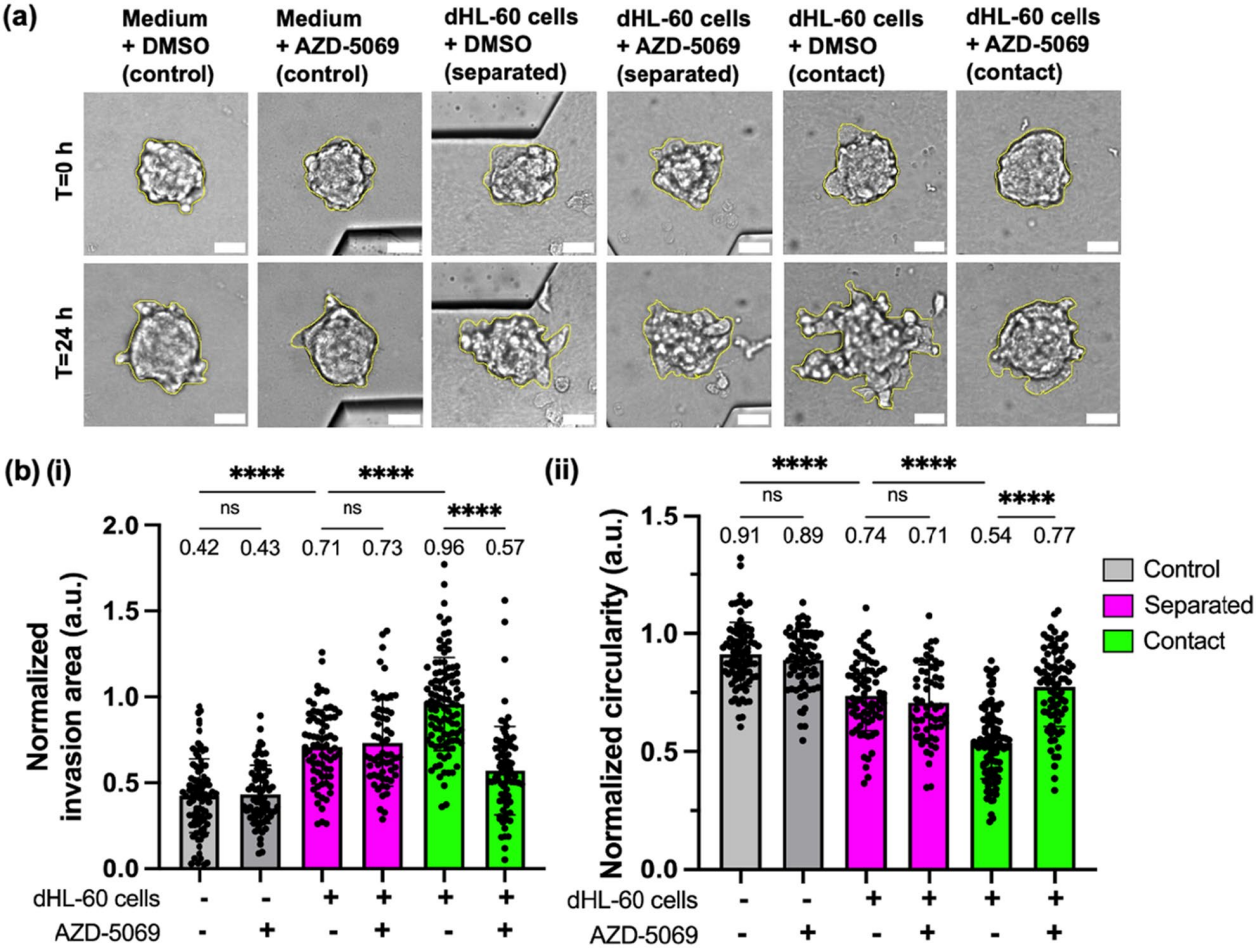
CXCR2 inhibition attenuates dHL-60 cell-promoted invasion of PANC-1 tumor spheroids in the “contact” scenario but not in the “separated” scenario. (**a**) Representative brightfield images showing invasion of tumor spheroids (boundary circled in yellow) into the surrounding collagen matrix at t = 0 h and 24 h after dHL-60 cells or culture medium ± 1 μM AZD-5069 were loaded into the central channel (“separated”) or along with tumor spheroids into the tumor gel channel (“contact”) of the NTI-chip. Scale bar, 50 µm. (**b**) Normalized invasion area (**i**), defined by the ratio of increase in the projected area of the invaded spheroids over 24 h to the initial projected area at t = 0 h, and normalized circularity (**ii**), defined by the ratio of spheroid circularity at t = 24 h to that at t = 0 h, in specified conditions. Each data point represents a spheroid and n = 57–100 spheroids per condition. Bars show mean ± SD with the mean values written above the points. At least three independent experiments were performed. ns: ≥ 0.05, ****: *p* < 0.0001, Brown-Forsythe and Welch ANOVA with Dunnett’s T3 multiple comparisons test.

We quantified spheroid invasion over 24 h in the NTI-chip by measuring the following two parameters: (1) normalized invasion area, defined as the difference between the final projected area of the spheroid (t = 24 h) and the initial projected area (t = 0 h) divided by the initial projected area^25,37,55–57^, and (2) normalized circularity, defined as the ratio of the final circularity of the spheroid (t = 24 h) to the initial circularity (t = 0 h)^58^. Since we found a negative correlation between the normalized circularity and the normalized invasion area of tumor spheroids undergoing spontaneous invasion in the gel matrix (Pearson *r* = − 0.3355, *p* = 0.0014) (Supplementary Fig. S8), we considered a lower normalized circularity as an indicator of greater invasion. We found that dHL-60 cells in the “separated” scenario increased tumor spheroid invasion by increasing the normalized invasion area from 0.42 to 0.71 (*p* < 0.0001) and decreasing the normalized circularity from 0.91 to 0.74 (*p* < 0.0001) compared to the culture medium control (Fig. 4a,b). This result suggests that dHL-60 cells can promote PANC-1 tumor spheroid invasion via release of soluble factors. We also found that dHL-60 cells induced greater tumor spheroid invasion in the “contact” scenario than in the “separated” scenario by increasing the normalized invasion area from 0.71 to 0.96 (*p* < 0.0001) and decreasing the normalized circularity from 0.74 to 0.54 (*p* < 0.0001), suggesting direct contact as a second tumor-promoting mechanism of dHL-60 cells (Fig. 4a,b). Taken together, these results show that dHL-60 cells promote invasion of PANC-1 tumor spheroids via both release of soluble factors and direct cell–cell contact.

### CXCR2 inhibition attenuates dHL-60 cell-promoted invasion of PANC-1 tumor spheroids in a contact-dependent manner

After discovering the inhibitory effect of AZD-5069 on dHL-60 cell migration toward PANC-1 tumor spheroids, we sought to examine whether AZD-5069 treatment of dHL-60 cells could have a reciprocal effect on PANC-1 tumor spheroid invasion. We found no significant difference in either normalized invasion area (*p* > 0.9999) or normalized circularity (*p* = 0.9878) between the AZD-5069 condition and DMSO vehicle control in the absence of dHL-60 cells, meaning that CXCR2 inhibition alone did not affect tumor spheroid invasion (Fig. 4a,b). Thus, any difference in tumor spheroid invasion observed between AZD-5069 treatment and DMSO vehicle control in the presence of dHL-60 cells would be mediated by AZD-5069 binding of dHL-60 cells, not PANC-1 cells.

We then assessed tumor spheroid invasion in the absence or presence of AZD-5069 (1 μM) in both “separated” and “contact” scenarios. We found that treatment of dHL-60 cells with AZD-5069 did not affect tumor spheroid invasion in the “separated” scenario, as there was no significant difference in either normalized invasion area (*p* > 0.9999) or normalized circularity (*p* = 0.9946) between the AZD-5069 condition and DMSO vehicle control (Fig. 4a,b). Strikingly, treatment of dHL-60 cells with AZD-5069 significantly reduced tumor spheroid invasion in the “contact” scenario by reducing the normalized invasion area from 0.96 to 0.57 (*p* < 0.0001) and increasing the normalized circularity from 0.54 to 0.77 (*p* < 0.0001) compared to DMSO vehicle control (Fig. 4a,b). Hence, CXCR2 inhibition via AZD-5069 attenuated dHL-60 cell-induced invasion of PANC-1 tumor spheroids only when they were allowed to engage in direct contact with dHL-60 cells. These results suggest that AZD-5069 may not block the release of tumor-promoting soluble factors by dHL-60 cells as effectively as it may block the direct contact between dHL-60 cells and tumor spheroids. These results also suggest that neutrophil receptors other than CXCR2 may be involved in the production and secretion of tumor-promoting soluble factors by neutrophils. The role of CXCR2 in controlling the secretome of neutrophils was not examined in this study and warrants further investigation.

### CXCR2 inhibition attenuates dHL-60 cell-promoted proliferation of PANC-1 tumor spheroids in a contact-dependent manner

**Figure 5:**
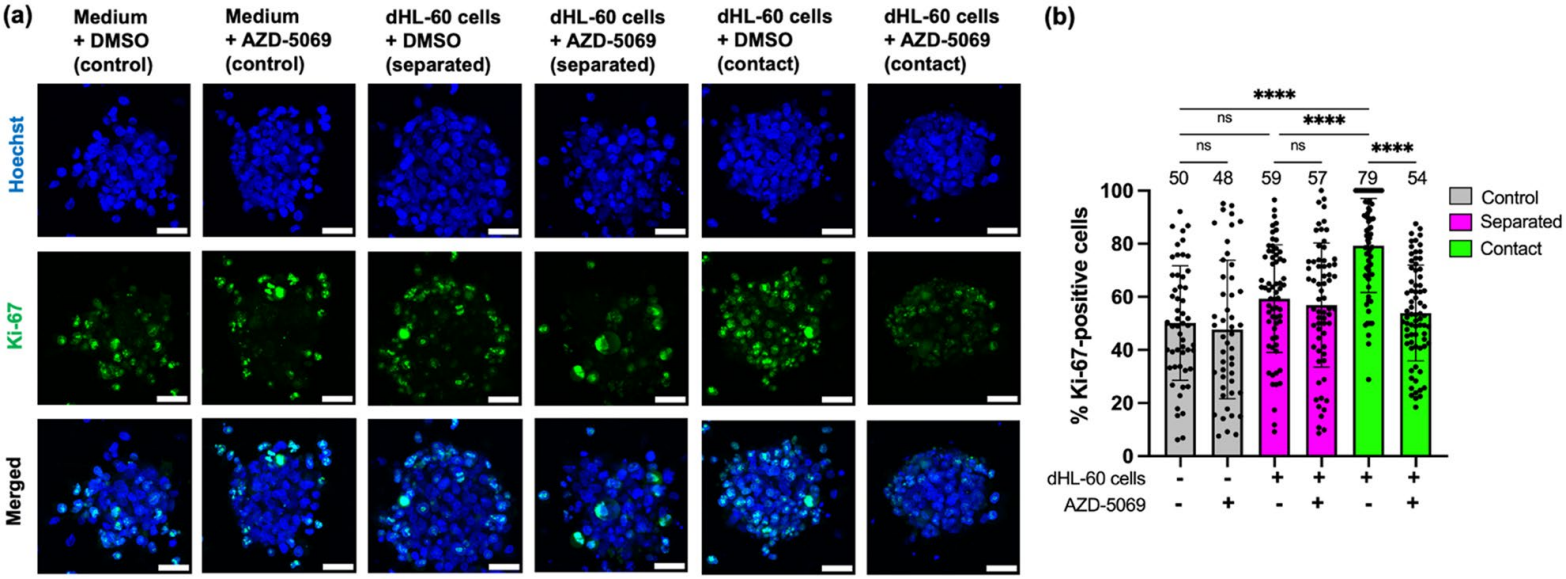
CXCR2 inhibition attenuates dHL-60 cell-promoted proliferation of PANC-1 tumor spheroids only in the “contact” scenario but not in the “separated” scenario. (**a**) Representative confocal images of PANC-1 tumor spheroids (blue, Hoechst) immunostained for Ki-67 (green), a proliferation marker, at t = 24 h after dHL-60 cells or culture medium ± 1 μM AZD-5069 were loaded into the central channel (“separated”) or along with tumor spheroids into the tumor gel channel (“contact”) of the NTI-chip. Images are maximum intensity projections of z-stacks with a 2 μm step size. Scale bar, 50 µm. (**b**) Bar plot showing the percentage of Ki-67-positive cells per spheroid in specified conditions. Each data point represents a spheroid and n = 53–73 spheroids per condition. Bars show mean ± SD with mean values written above the points. At least three independent experiments were performed. ns: *p* ≥ 0.05, ****: *p* < 0.0001, Brown-Forsythe and Welch ANOVA with Dunnett's T3 multiple comparisons test.

When tumor spheroids undergo invasion in the hydrogel, cancer cells in the spheroids could also undergo proliferation at the same time^37,59^. Such proliferation could increase the volume and thus the projected area of the spheroid. Hence, the increase in the spheroid invasion area induced by dHL-60 cells in both “separated” and “contact” scenarios (Fig. 4) may at least partially reflect an upregulation in proliferation. Similarly, the reduction in the spheroid invasion area by AZD-5069 treatment in the “contact” scenario (Fig. 4) may also partially reflect a downregulation in proliferation. To further unravel proliferation as a potential underlying cause of the observed changes in spheroid invasion area, we examined the expression of proliferation marker Ki-67^60^ by tumor spheroids via immunofluorescence after 24 h of time-lapse imaging. The proliferation level of PANC-1 tumor spheroids was quantified as the percentage of proliferating cells (i.e., cells that stained positive for Ki-67) per spheroid. We first confirmed that dHL-60 cells do not express Ki-67 using immunofluorescence and thus that the presence of dHL-60 cells will not affect the measurement of the proliferation level of tumor spheroids in the “contact” scenario (Supplementary Fig. S9). We found no significant difference in the proliferation level between the AZD-5069 condition and DMSO vehicle control in the absence of dHL-60 cells (50% vs. 48%, *p* > 0.9999), meaning that CXCR2 inhibition alone did not affect the proliferation of tumor spheroids (Fig. 5a,b). This result corroborates a previous study reporting that CXCR2 inhibition does not affect in vitro proliferation of mouse pancreatic cancer cells^20^. Thus, any observed difference in tumor spheroid proliferation between AZD-5069 treatment and DMSO vehicle control in the presence of dHL-60 cells would be mediated by AZD-5069 binding of dHL-60 cells, not PANC-1 cells.

We found that dHL-60 cells in the “separated” scenario did not significantly increase the proliferation level of spheroids compared to the culture medium control (59% vs. 50%, *p* = 0.2509) (Fig. 5a,b), suggesting that soluble factors released by dHL-60 cells did not promote the proliferation of PANC-1 tumor spheroids. In the “contact” scenario, dHL-60 cells significantly increased the proliferation level of spheroids compared to both culture medium control (79% vs. 50%, p < 0.0001) and dHL-60 cells in the “separated” scenario (79% vs. 59%, *p* < 0.0001) (Fig. 5a,b), suggesting that dHL-60 cells can enhance the proliferation of PANC-1 tumor spheroids via direct contact. Although AZD-5069 treatment did not affect the proliferation level of spheroids in the “separated” scenario (59% vs. 57%, p > 0.9999) (Fig. 5a,b), we found a significant reduction in the proliferation level of spheroids from 79 to 54% (*p* < 0.0001) by AZD-5069 treatment in the “contact” scenario (Fig. 5a,b), meaning that the attenuation of tumor spheroid proliferation by CXCR2 inhibition is dependent on contact with dHL-60 cells. Together with results from Fig. 4, these results suggest that direct contact between neutrophils and cancer cells may be required for CXCR2 inhibition to suppress tumor invasion and proliferation.

### CXCR2 inhibition reduces frequency and duration of dHL-60 cell contact with PANC-1 tumor spheroids

**Figure 6:**
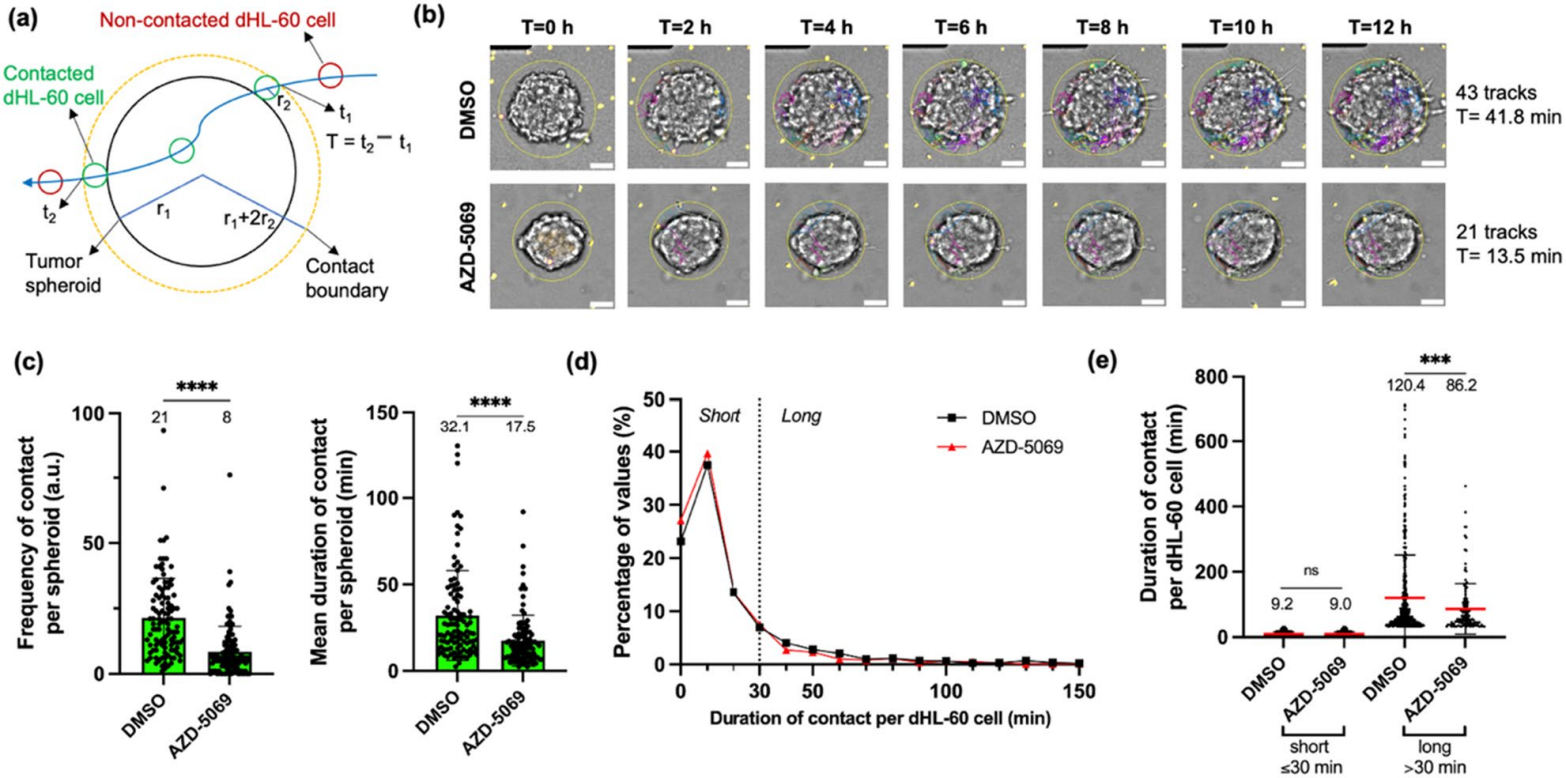
CXCR2 inhibition reduces frequency and duration of direct contact of dHL-60 cells with PANC-1 tumor spheroids in the “contact” scenario. (**a**) A schematic showing the definition of contact between a migrating dHL-60 cell (track shown in blue) and a given tumor spheroid. r_1_ = radius of the tumor spheroid, r_2_ = radius of a dHL-60 cell. T = duration of contact. Black = boundary of the tumor spheroid, yellow = boundary of the circular “contact” region with a radius of r_1_ + 2 * r_2_, green = dHL-60 cells in contact, red = dHL-60 cells not in contact. (**b**) Representative images showing dHL-60 cells (yellow) treated with DMSO vehicle control or 1 μM AZD-5069 interacting with PANC-1 tumor spheroids (brightfield) at t = 0 h, 2 h, 4 h, 6 h, 8 h, 10 h, and 12 h of time-lapse imaging in the “contact” scenario, overlaid with dHL-60 cell tracks extracted by TrackMate (ImageJ). Any dHL-60 cells that entered the circular ROI (yellow) around the spheroid are considered to be in contact with the spheroid. Scale bar, 50 µm. (**c**) Bar plots showing the frequency (left panel) and mean duration (right panel) of contact with dHL-60 cells per tumor spheroid over 12 h in specified conditions. Each data point represents a spheroid and n = 107–119 spheroids per condition. Bars show mean ± SD with mean values written above the points. ****: *p* < 0.0001, unpaired t test with Welch’s correction. (**d**) Distributions of durations of contact per dHL-60 cell in DMSO vehicle control and AZD-5069 treated conditions. Only contacts shorter than 150 min are shown. (**e**) Contact between a dHL-60 cell and a tumor spheroid was classified into two categories: short (≤ 30 min) and long (> 30 min). Scatter plot showing the duration of contact per dHL-60 cell in specified conditions. Each data point represents a dHL-60 cell and n = 133–461 cells per condition in long contact. Bars show mean (red) ± SD (black) with mean values written above the points. At least three independent experiments were performed. ns: ≥ 0.05, ***: *p* < 0.001, unpaired t test with Welch’s correction.

Based on findings in Figs. 4 and 5, we hypothesize that CXCR2 inhibition attenuates tumor spheroid invasion and proliferation by blocking direct contact with dHL-60 cells. To test this hypothesis, we examined the temporal dynamics of contact between dHL-60 cells and tumor spheroids in the absence or presence of AZD-5069 in the “contact” scenario. Specifically, for each tumor spheroid, we measured both the frequency and duration of contact with the surrounding dHL-60 cells in the first 12 h of time-lapse imaging. In this study, contact is defined as a situation in which the distance between the center of a tumor spheroid and the center of a dHL-60 cell is less than the sum of the radius of the tumor spheroid and the diameter of the dHL-60 cell^61,62^ (Fig. 6a). The frequency of contact is the number of times that a given tumor spheroid formed contact with any dHL-60 cell. The duration of contact is the length of time for which the contact between each dHL-60 cell and the tumor spheroid lasted. We first examined the mean duration of contact averaged for all dHL-60 cells per tumor spheroid. We found that AZD-5069 (1 μM) significantly reduced both the frequency of contact from 21 to 8 (*p* < 0.0001) and the mean duration of contact from 32.1 min to 17.5 min (*p* < 0.0001) per spheroid compared to DMSO vehicle control (Fig. 6b,c; Supplementary Videos 1 and 2). To further probe the contacts with single-neutrophil resolution, we then analyzed the duration of contact per dHL-60 cell instead of the mean values per spheroid. The frequency distributions of durations of contact per dHL-60 cell with and without AZD-5069 show that AZD-5069 reduced the frequency of durations of contact longer than 30 min (Fig. 6d). Thus, we classified all contacts into two arbitrary categories: short contacts (≤ 30 min) and long contacts (> 30 min). AZD-5069 (1 μM) did not affect the duration of short contact per dHL-60 cell (9.2 min vs. 9.0 min, *p* = 0.89) (Fig. 6e), possibly because short contacts may include random collisions and CXCR2-independent contacts. However, the drug treatment significantly reduced the duration of long contact from 120.4 min to 86.2 min (*p* = 0.0002) per dHL-60 cell (Fig. 6e). These results further corroborate our findings in Figs. 4 and 5 and more importantly reveal blockade of long contact with neutrophils as a novel mechanism by which CXCR2 inhibition attenuates tumor invasion and proliferation.

Moreover, we found that AZD-5069 treatment significantly reduced the number of tumor-infiltrated dHL-60 cells from 2.1 cells to 1.3 cells per spheroid compared to DMSO vehicle control at t = 24 h in the “contact” scenario (n = 30–39 spheroids per condition, *p* = 0.0058) (Supplementary Fig. S10). This result shows that CXCR2 inhibition can also reduce the infiltration of dHL-60 cells into tumor spheroids in addition to direct contact with them. Interestingly, the number of infiltrated dHL-60 cells per spheroid was low in both DMSO (2.1 ± 1.3) and AZD-5069 (1.3 ± 1.0) conditions. The overall low level of neutrophil infiltration in tumor spheroids in this study could be partially due to the intrinsic difference in functionality between dHL-60 cells, the neutrophil-like cell line, and primary human neutrophils^63^. We could promote neutrophil infiltration into tumor spheroids in a future study by using primary neutrophils isolated from human blood or stimulating tumor spheroids with inflammatory cytokines such as tumor necrosis factor-α (TNF-α)^24^ prior to interaction with dHL-60 cells.

## Discussion

In this work, we engineered a human cell-based NTI-chip that enabled us to decipher the tumor-promoting mechanisms of neutrophils and the tumor-suppressive mechanisms of CXCR2 inhibition as a novel neutrophil-based immunotherapy in pancreatic cancer. The freedom to position neutrophils either in direct contact with or separated from cancer cells in our system allows us to reveal the importance of direct contact in addition to soluble factors. Our system enables multi-parametric generation of quantitative information about cellular phenotypes including neutrophil migration (forward migration index, velocity, directionality, and displacement), spheroid invasion (area and circularity), and neutrophil-spheroid contact (frequency and duration). The detailed temporal information of neutrophil-cancer interactions obtained in this work adds a valuable dimension to the landscape of classic immunology research dominated by standard end-point molecular biology assays^26,45^. In addition, we demonstrated the utility of our microphysiological system to perform end-point molecular biology assays such as immunofluorescence staining to examine expressions of protein markers (CXCR2 and Ki-67) in situ and ELISA to examine cytokine secretion (IL-8) from collected supernatant.

Besides mechanistic investigation of cell–cell interactions, this study also has critical clinical implications. We picked AZD-5069, a small molecule CXCR2 antagonist, to test in our platform because it was shown to not adversely affect the mobilization of neutrophils from bone marrow into the peripheral circulation or key antimicrobial immune functions of neutrophils in healthy human volunteers^64^. AZD-5069 was also shown to be well tolerated in phase II clinical trials for respiratory diseases^65–67^ and is currently being assessed in clinical trials for the treatment of prostate cancer, head and neck squamous cell carcinoma, and pancreatic ductal carcinoma^22^. In the current study, we found that AZD-5069 treatment effectively reduces human neutrophil migration toward pancreatic tumor spheroids, suggesting the potential of AZD-5069 to block the recruitment of neutrophils to the tumor tissue in pancreatic cancer patients and thereby prevent more neutrophils from entering the tumor tissue, becoming TANs, and then performing tumor-promoting functions. More importantly, we found that AZD-5069 treatment attenuates invasion and proliferation of pancreatic tumor spheroids in a neutrophil contact-dependent manner and significantly reduces direct contact between neutrophils and tumor spheroids. These findings suggest that AZD-5069 might be able to suppress tumor progression in pancreatic cancer patients by blocking direct contact between TANs and cancer cells in the tumor tissue. Hence, our study indicates that AZD-5069 could have a therapeutic effect by affecting not only circulating neutrophils but also TANs. Therefore, it may be important clinically to employ special drug delivery platforms, routes of administration, and formulations of AZD-5069 to target TANs which are located inside the tumor tissue, as opposed to only targeting circulating neutrophils in blood, to maximize its therapeutic potential. Thus, the findings of this study will inspire the design, development, and optimization of more subtle, delicate, restrained, and complex neutrophil-based immunotherapy strategies^2^.

One limitation of this study is that neutrophils were embedded in collagen gel in the “contact” scenario but suspended in culture medium in the “separated” scenario, which renders the potential effect of collagen gel on neutrophil phenotypes unknown. While we needed the gel-liquid interface as a barrier to keep the separated nature of the scenario in our current design, a more advanced system could be developed in the future where neutrophils can be embedded in collagen gel and spatially separated from tumor spheroids at the same time. We could also engineer a more biomimetic system by building an endothelial lumen around the central channel to assess the effect of CXCR2 inhibition on neutrophil extravasation into the tumor tissue^68–72^. The choice of not including endothelial cells in our system was intentional as previous studies show that neutrophils are unable to migrate into type I collagen gel without the presence of endothelial cells between neutrophils and the gel^43,44^. Thus, the incorporation of endothelial cells is expected to promote neutrophil transmigration from the central channel into the tumor gel channel in our system, which would eliminate the “separated” scenario. To confirm the involvement of CXCR2 in neutrophil-tumor interactions, further experiments can be performed to inhibit the CXCR2 pathway using other methods than pharmacological antagonism (AZD-5069), such as knockout of CXCR2 in HL-60 cells. To better mimic the tumor microenvironment of cancer patients where neutrophils are already present within the tumor tissue before drug treatment, we can grow heterotypic tumor spheroids by mixing PANC-1 cancer cells and dHL-60 cells together at the beginning of spheroid culture and then assess drug efficacy in a future study. Furthermore, we used immortalized human cell lines only in this study, which limits its clinical relevance. The NTI-chip developed in this study will enable future research with clinical samples (a few microliters)^61^, allowing for the measurement of interactions between patient-derived tumor cells and autologous neutrophils. This, combined with the presence of drug candidates and precise spatiotemporal control, opens up avenues for personalized medicine^73^. With the US Food and Drug Administration (FDA) approval of the first clinical trial based solely on organ-on-a-chip models in July 2022 and the US Senate passing of the FDA Modernization Act 2.0 adding “organ chips and microphysiological systems” to the list of nonclinical tests in September 2022^74^^,75^, it is now possible for microphysiological systems to serve as a crucial complementary tool to animal models for both mechanistic studies and preclinical testing of novel neutrophil-based cancer immunotherapies.

## Methods

### Cell culture

The human promyelocytic leukemia cell line HL-60 (American Type Culture Collection ATCC, CCL-240) was cultured in Iscove’s Modified Dulbecco’s Medium (IMDM, ATCC) supplemented with 20% fetal bovine serum (FBS, ATCC). HL-60 cells from passages 2 to 14 at a density of 1.5 × 10^5^ cells/mL were differentiated into a neutrophil-like state for 5 days after adding 1.5% dimethyl sulfoxide (DMSO, ATCC) (denoted as dHL-60 cells)^45,46,76^. The human pancreatic cancer cell line PANC-1 (ATCC, CRL-1469) was cultured in Dulbecco’s Modified Eagle’s Medium (DMEM, ATCC) supplemented with 10% FBS and 1% penicillin–streptomycin (Gibco). For passaging, PANC-1 cells were detached using TrypLE™ Express Enzyme (Gibco) for 4 min at 37 °C with 5% CO_2_. PANC-1 cells were harvested from passages 2 to 13 for 3D tumor spheroid culture. All cell cultures were maintained at 37 °C with 5% CO_2_.

### 3D tumor spheroid culture

3D PANC-1 tumor spheroids were generated using the Elplasia™ round-bottom ultra-low attachment (ULA) 24-well plate (Corning, 4441) according to the manufacturer’s protocol. Briefly, six wells on the plate were each prewetted with 500 µL of complete DMEM and centrifuged to remove air bubbles from the microcavities. Each well was then seeded with 1 mL of ~ 16,620 PANC-1 cells (~ 30 cells per microcavity, 554 microcavities per well). The plate was then incubated at 37 °C with 5% CO_2_ for 5 days to form one spheroid in each microcavity. The plate was imaged using the brightfield channel of a Nikon ECLIPSE Ti2-E microscope (Plan Apo 10X objective, NA = 0.45) on day 5 to measure the diameters of the tumor spheroids. The diameter was calculated by averaging the longest and shortest axes of the spheroid.

### Design and fabrication of the NTI-chip

The NTI-chip comprises a central channel for loading dHL-60 cells or culture medium (9 mm long, 1 mm wide), a tumor gel channel for loading hydrogel containing PANC-1 tumor spheroids ± dHL-60 cells (9 mm long, 0.7 mm wide), an empty gel channel for loading empty hydrogel (9 mm long, 0.7 mm wide), and two medium channels, each having two medium reservoirs for loading culture medium (6.7 mm long, 1 mm wide) (Fig. 1a). The schematic in Fig. 1a was drawn in Rhino 7 and Biorender.com. The five parallel channels are approximately 164 µm in height and interconnected by four arrays of 10 trapezoidal posts (base lengths: 300 µm and 185 µm, height: 100 µm, distance between posts: 100 µm) that enable the confinement of hydrogel through a balance between surface tension and capillary forces.

The master mold was fabricated using standard photolithography techniques. Briefly, the design of the NTI-chip was drawn in AutoCAD (Autodesk) and printed onto a chrome-on-glass dark-field photomask. A silicon wafer (University Wafer) was spin-coated with negative photoresist SU-8 50 (Kayaku Advanced Materials) at 1000 rpm for 30 s and soft-baked at 65 °C for 10 min and 95 °C for 2.5 h. The silicon wafer was exposed to ultraviolet (UV) light through the photomask at 450 mJ/cm^2^. The exposed wafer was then baked at 65 °C for 1 min and 95 °C for 10 min and developed with SU-8 developer for 16 min. Lastly, the height of the features on the wafer was measured by a profilometer.

NTI-chips were fabricated using standard soft lithography techniques. Briefly, polydimethylsiloxane (PDMS) and the curing agent (Sylgard 184, Dow Corning) were mixed thoroughly at a 10:1 ratio, degassed in a desiccator for 1 h, poured over the master mold, and cured in an oven at 65 °C overnight. Solidified PDMS was then peeled from the master mold and individual chips were cut out. The inlets and outlets of the two medium channels were punched using a 4 mm biopsy puncher (Ted Pella Inc.) to create medium reservoirs and those of the two gel channels and the central channel using a 1 mm biopsy puncher. Following air plasma treatment (Harrick Plasma) at high radio frequency (RF) power for 2 min, six chips were bonded to a glass-bottom 6-well plate (Cellvis, P061.5HN) and placed on a hotplate at 80 °C for 2 h to strengthen bonding.

The NTI-chips were sterilized under UV light in a laminar flow hood for 30 min. The gel channels and central channels were coated with human fibronectin (10 µg/mL in sterile deionized water) (Sigma-Aldrich) at 37 °C for 1 h to promote attachment of collagen gel to the channel surfaces and attachment of dHL-60 cells to the glass substrate. The channels were then rinsed with sterile deionized water twice and dried on a hotplate at 80 °C for 24 h to restore hydrophobicity^77^. Methods of the dextran diffusion assay and testing fluid flow in the NTI-chip are described in Supplementary Methods.

### Neutrophil-tumor spheroid interactions in the NTI-chip

dHL-60 cells were collected on day 5 of differentiation, stained with 20 µM CellTracker Orange CMRA dye (Invitrogen, C34551) at 37 °C for 30 min, and pre-treated with 1 µM CXCR2 antagonist AZD-5069 (MedChemExpress LLC) or DMSO vehicle control in complete IMDM on a rotator at 37 °C for 30 min. PANC-1 tumor spheroids were collected from the ULA 24-well plate on day 5 of 3D culture and filtered through a 150 µm cell strainer (pluriSelect) to obtain spheroids with diameters smaller than 150 µm to prevent big spheroids from clogging the microfluidic channels in the NTI-chip. All pipette tips used to transfer tumor spheroids were pre-coated with 2% bovine serum albumin** (**BSA, Sigma-Aldrich) to reduce spheroid loss. To acquire representative confocal images of “separated” and “contact” scenarios, tumor spheroids were stained with 20 µM CellTracker Blue CMAC dye (Invitrogen, C2110) at 37 °C for 30 min. To prepare a 2.5 mg/mL collagen gel solution with a pH of 7.4, 4.39 mg/mL rat-tail type I collagen gel (Corning, 354,236) was diluted in a mixture of 10× phosphate-buffered saline (PBS, Gibco), sterile deionized water, and 0.5 N NaOH (Fisher Chemical) on ice^77,78^. The final pH of the gel solution was confirmed to be approximately 7.4 using a pH indicator paper.

To create the “separated” scenario, ~ 10–20 tumor spheroids in 6 µL of collagen gel solution were loaded into the tumor gel channel of the NTI-chip on ice. To create the “contact” scenario, ~ 10–20 tumor spheroids and ~ 12,000 dHL-60 cells (± 1 μM AZD-5069) in 6 µL of collagen gel solution were loaded together into the tumor gel channel of the NTI-chip on ice. In both scenarios, 6 µL of empty collagen gel solution was loaded into the empty gel channel on ice as a negative control. The NTI-chips were incubated at 37 °C with 5% CO_2_ for 40 min to polymerize the gel. The two medium channels and the central channel were then filled with 100 µL and 8 µL of complete IMDM (± 1 µM AZD-5069) respectively to keep the NTI-chips hydrated. The NTI-chips were incubated at 37 °C with 5% CO_2_ for 20 min to stabilize any initial fluctuations caused by small differences in the volume of medium in each medium reservoir. For the “separated” scenario, ~ 12,000 dHL-60 cells (± 1 μM AZD-5069) in 8 µL of complete IMDM were loaded into the central channel after stabilization. The gel barriers prevent dHL-60 cells from migrating into the gel channels and help maintain the “separated” nature of the scenario^43,44^.

To create the positive control condition for migration of dHL-60 cells in the NTI-chip, 6 µL of empty collagen gel solution was loaded into both gel channels on ice. After 40 min gel polymerization, the lower medium channel was filled with 100 µL complete IMDM. The upper medium channel was filled with 100 µL of human IL-8 solution (10 nM in complete IMDM) (Miltenyi Biotec, 130–122-353), a known chemoattractant for neutrophils^28,50^. Then, the central channel was filled with 8 µL of complete IMDM. After 20 min stabilization, ~ 12,000 dHL-60 cells in 8 µL of complete IMDM were loaded into the central channel. The method of the transwell migration assay to assess the effect of AZD-5069 alone on dHL-60 cell migration is described in Supplementary Methods.

### Time-lapse Imaging and analysis

Immediately after seeding dHL-60 cells, time-lapse imaging of six NTI-chips was performed concurrently on a fully automated Nikon ECLIPSE Ti2-E microscope ﻿using a Plan Apo 10X objective (NA = 0.45) at 37 °C with 5% CO_2_ for 24 h to capture migration of dHL-60 cells in the “separated” scenario, dynamics of contact between dHL-60 cells and tumor spheroids in the “contact” scenario, and invasion of tumor spheroids into the surrounding gel matrix in both “separated” and “contact” scenarios. Images were acquired using NIS-elements (Nikon Inc.) software and recorded using brightfield and TRITC channels at 2.5-min intervals for the first 6 h (“separated”) or 12 h (“contact”) and at 1 h intervals for the remaining 18 h (“separated”) or 12 h (“contact”) to minimize photobleaching over time. For experiments involving IL-8 positive control, time-lapse imaging was performed at 2.5 min intervals for 6 h in total.

To quantify dHL-60 cell migration from 0 to 6 h, automatic cell tracking was performed on TRITC images using the TrackMate plugin in ImageJ (National Institutes of Health)^62,79^. The raw positional data exported from TrackMate were further analyzed using the Chemotaxis and Migration Tool plugin (Ibidi) in ImageJ to generate trajectory plots and calculate the following parameters of cell migration: forward migration index in the y-direction (y-FMI), mean velocity, maximum velocity, displacement (Euclidean distance), and directionality (Table 1)^51^. Notably, y-FMI is defined as the y-coordinate of the ending position of a given track after setting the starting position as (0,0) divided by the accumulated distance and has a range of values from − 1 to 1^51^. Since the chemoattractant source (tumor spheroids or IL-8 solution) is located above dHL-60 cells in microscopic images of the NTI-chip, y-FMI measures the efficiency of dHL-60 cell migration toward tumor spheroids or IL-8. In contrast, directionality is defined as the ratio of the Euclidean distance and the accumulated distance between the starting and the ending positions of a given track, has a range of values from 0 to 1, and only measures the straightness of cell motion with no regard to the relative location of the chemoattractant source^51^. Only tracks whose mean velocity was higher than 0.01 µm/s were used for data analysis to exclude non-migratory cells.

To quantify invasion of tumor spheroids into the surrounding gel matrix from 0 to 24 h, the boundaries of each tumor spheroid at t = 0 h and t = 24 h were manually drawn using brightfield images in ImageJ^25,37,55,56,58^. Both the area and the circularity of the circled region were measured in ImageJ. The normalized invasion area of a spheroid was calculated as the difference between the final projected area (t = 24 h) and the initial projected area (t = 0 h) divided by the initial projected area. The normalized circularity of a spheroid was calculated as the ratio of the final circularity (t = 24 h) and the initial circularity (t = 0 h). Circularity is defined by the following formula: 4π*area/perimeter^2 and has a range of values from 0 to 1. As the circularity of tumor spheroids usually decreases when they invade and protrude into the surrounding matrix, circularity was also included as a quantitative indicator of invasion in addition to invasion area^58^.

To quantify neutrophil-tumor spheroid contact from 0 to 12 h, a circular region of interest (ROI) was defined around a certain tumor spheroid using brightfield images in ImageJ^61,62,80^. The diameter of the ROI equals the sum of the measured long-axis diameter of the tumor spheroid at t = 0 h and twice the diameter of an average dHL-60 cell (13 µm) (Fig. 6a). The center of the ROI was manually placed at the approximate center of the tumor spheroid at t = 0 h. Any dHL-60 cells that entered this ROI were considered to be in contact with the tumor spheroid. Automatic tracking of dHL-60 cells in the circular ROI was performed on TRITC images from 0 to 12 h using TrackMate. The number of detected tracks is the frequency or the number of times that a dHL-60 cell formed contact with the tumor spheroid. The duration of each track is the duration of contact between each dHL-60 cell and the tumor spheroid. The mean duration of contact per tumor spheroid was calculated by averaging durations of all tracks per ROI.

### Immunofluorescence staining, confocal imaging, and analysis

PANC-1 tumor spheroids in the NTI-chip were stained for Ki-67, a proliferation marker, to assess their proliferation level in separate experiments. CXCR2 expressions by dHL-60 cells and PANC-1 tumor spheroids were also assessed. Immunofluorescence staining in the NTI-chip was performed as previously described^77^. Briefly, after 24 h of time-lapse imaging, cells were washed with PBS (Gibco) three times and fixed with 4% paraformaldehyde (Electron Microscopy Sciences) for 15 min at room temperature. The cells were then washed with PBS twice and permeabilized with 0.1% Triton X-100 (Thermo Scientific Chemicals) for 1 h at room temperature. Permeabilization was skipped for immunofluorescence of CXCR2 to avoid intracellular staining. Cells were washed with PBS twice and blocked with 10% goat serum (Invitrogen, 50062Z) for 2 h at room temperature. The NTI-chips were then incubated overnight at 4 °C with one of the following primary antibodies diluted in 10% goat serum: mouse anti-Ki-67 (1:1000, Invitrogen, 14,569,980) or rabbit anti-CXCR2 (1:200, Invitrogen, PA5-100,951). The NTI-chips were washed with PBS twice and incubated with PBS for 24 h at 4 °C on a rocker for thorough washing. The NTI-chips were incubated for 2 h at room temperature with the goat anti-mouse Alexa Fluor 488 secondary antibody (1:1000, Invitrogen, A11001) or goat anti-rabbit Alexa Fluor 488 secondary antibody (1:1000, Invitrogen, A11008) and nuclei stain Hoechst 33,342 (1:1000, Thermo Scientific, 62,249) diluted in 10% goat serum, washed with PBS twice, and incubated with PBS for 24 h at 4 °C on a rocker for thorough washing. All reagents were added to and removed from the NTI-chips through the medium reservoirs in the volume of 100 µL. Immunofluorescence staining of Ki-67 in HL-60 cells and dHL-60 cells is described in Supplementary Methods.

The NTI-chips were then imaged with a confocal laser scanning microscope (Olympus FV3000RS). The UPlanSApo 20X air objective (NA = 0.75) was used for imaging NTI-chips with tumor spheroids and the UPlanSApo 40X silicone oil objective (NA = 1.25) for imaging NTI-chips with only dHL-60 cells. Z-stacks of tumor spheroids were acquired at 2 µm intervals using FLUOVIEW software and covered a thickness of 100–160 µm. To quantify Ki-67 expression as the percentage of Ki-67-positive cells per spheroid, the number of Ki-67-positive cells was normalized by the total number of cells per spheroid. The total number of cells per spheroid was measured based on Hoechst signals using the spot function of Imaris 10.0.0 software (Oxford Instruments). The estimated diameter of the cell nuclei was manually optimized to be 12 µm for accurate spot detection. The number of Ki-67-positive cells was measured based on FITC signals using the spot function of the software. To quantify dHL-60 cell infiltration per tumor spheroid, the boundary of each tumor spheroid was first manually drawn using brightfield images in ImageJ^25^. The number of dHL-60 cells (CMRA-stained) located inside the boundary was manually counted in ImageJ based on TRITC signals. ImageJ was used to create representative confocal images using maximum intensity projections of z-stacks and apply the same brightness and contrast settings to images of all experimental conditions for each channel.

### Flow cytometry

HL-60 cells were collected before differentiation to examine CD11b expression. dHL-60 cells were collected on day 5 of differentiation to examine CD11b and CXCR2 expressions. To avoid digestion of surface proteins by trypsin, PANC-1 cells in 2D culture were detached using TrypLE™ Express Enzyme for 4 min at 37 °C with 5% CO_2_ and collected to examine CXCR2 expression^81^. PANC-1 tumor spheroids were dissociated into single cells using TrypLE™ Express Enzyme for 10 min at 37 °C with 5% CO_2_ and collected to examine CXCR2 expression. All cells were washed with PBS once and stained with Zombie UV™ Fixable Viability Kit (BioLegend, 423107) to label dead cells for 30 min at room temperature. After washing with Flow Cytometry Staining Buffer (Invitrogen, 00422226) once, cells were stained with the corresponding fluorescently conjugated antibodies anti-human CD11b-Alexa Fluor 488 (BioLegend, 301317) and anti-human CXCR2-PE (BioLegend, 320706) diluted in Flow Cytometry Staining Buffer for 30 min at 4 °C. After washing with Flow Cytometry Staining Buffer once, cell samples were run on an LSR Fortessa flow cytometer (BD Biosciences). Data were analyzed using FlowJo 10.8.1 software (BD). For the gating strategy, cells were first gated based on the side scatter area (SSC-A, cell granularity) versus forward scatter area (FSC-A, cell size) density plot to exclude debris. Single cells were then gated based on the forward scatter height (FSC-H) versus forward scatter area (FSC-A) density plot to exclude cell doublets. Live cells were then gated based on their negative staining for the Zombie dye to exclude dead cells.

### Enzyme-linked immunosorbent assay (ELISA)

The supernatant (conditioned media) was collected from the ULA 24-well plate on day 5 of 3D tumor spheroid culture. The spheroids were then suspended in collagen gel and loaded into both gel channels of the NTI-chip as described in the previous section “Neutrophil-Tumor Spheroid Interactions in the NTI-chip”. The spheroids were cultured in complete DMEM in the NTI-chip for 5 days. On day 3, 30 µL of complete DMEM was added to each medium channel to compensate for evaporation. The supernatant was collected from the medium reservoirs of the NTI-chip on day 5. All supernatants were centrifuged at 2000×*g* for 10 min at 4 °C to remove cellular debris. All supernatants were frozen at − 80 °C for up to one month until analyzed. Concentrations of IL-8 in supernatants were measured using the human IL-8 ELISA kit (BioLegend, 431507) according to the manufacturer’s protocol. Samples were tested in triplicate or duplicate without dilution. A Synergy H4 hybrid microplate reader (BioTek) was used to read the absorbance at 450 nm. Prism 9 software (GraphPad) was used to generate standard curves and perform data interpolation.

### Statistical analysis

All experiments were performed and replicated at least three times, unless otherwise stated. Statistical analysis was performed using Prism 9 software (GraphPad). Data were expressed as means  ± standard deviations. All statistical tests used for the experiments can be found in the corresponding figure legends. Differences were considered statistically significant for *p* < 0.05.

### Supplementary Information

Supplementary Information 1.

Supplementary Video 1.

Supplementary Video 2.

## Data Availability

The datasets generated and analyzed during the current study are available from the corresponding author C.N.J. on reasonable request.
