# Supplementary material for: A microphysiological system reveals neutrophil contact-dependent attenuation of pancreatic tumor progression by CXCR2 inhibition-based immunotherapy
Source: Sci Rep. 2024 Jun 19;14:14142. doi: 10.1038/s41598-024-64780-4 (PMC11187156; doi:10.1038/s41598-024-64780-4)
Supplement: Supplementary file 1 — Supplementary Information 1. [file 41598_2024_64780_MOESM1_ESM.docx]

**Supplementary Information**

**A microphysiological system reveals neutrophil contact-dependent attenuation of pancreatic tumor progression by CXCR2 inhibition-based immunotherapy**

**Shuai Shao^1 2^, Nikki A. Delk^3^, Caroline N. Jones^1 2 *^**

^1^ Department of Bioengineering, The University of Texas at Dallas, Richardson, TX, 75080, USA

^2^ Department of Biomedical Engineering, UT Southwestern Medical Center, Dallas, TX, 75235, USA

^3^ Department of Biological Sciences, The University of Texas at Dallas, Richardson, TX, 75080, USA

**^*^** email: caroline.jones@utdallas.edu

**Supplementary Results**

**Validation of CD11b expression by dHL-60 cells**

Since HL-60 cells demonstrate a neutrophil-like phenotype only after differentiation ^1–3^, we validated the success of our differentiation protocol by examining expression levels of neutrophil surface marker CD11b ^4,5^ by HL-60 cells before and after differentiation using flow cytometry. We found that CD11b was lowly expressed by HL-60 cells before differentiation (% CD11b-positive cells = 12.2 ± 2.4) but highly expressed by dHL-60 cells after differentiation (% CD11b-positive cells = 95.9 ± 0.6) (p < 0.001) **(Fig. S3)**.

**Characterization and validation of tumor spheroid phenotypes in the NTI-chip**

Due to the height limit of the microfluidic channels (~164 µm), we filtered the collected spheroids through a 150 µm cell strainer to exclude big spheroids (diameter > 150 µm) before loading them into the NTI-chip. The average diameter of spheroids was significantly reduced from 124.2 µm to 107.4 µm before and after filtering (p < 0.0001) **(Fig. S4a)**. Due to the technical difficulty in loading the same number of spheroids into each chip, we characterized the frequency distribution of the number of spheroids loaded per chip in the “separated scenario” **(Fig. S4b)**. Since the chip-to-chip variability of the spheroid number may lead to varying degrees of neutrophil migration in the “separated” scenario, we decided to only analyze neutrophil migration in chips with 10 to 17 spheroids (25% to 75% percentiles) for assessment of the drug effect on neutrophil migration **(Fig. S4b)**.

Using ELISA, we validated the ability of PANC-1 tumor spheroids both on the ULA plate and in the NTI-chip to secrete human interleukin-8 (IL-8), a chemokine and CXCR2 ligand known to induce neutrophil chemotaxis ^6,7^ and the main chemokine released by the tumor tissue to recruit neutrophils in vivo ^8,9^ **(Fig. S4ci)**. We also found that the mean quantity of IL-8 secreted per spheroid was not statistically different between spheroids on the ULA plate and those in the NTI-chip over 5 days (0.145 vs. 0.149 pg, p= 0.4369), meaning that our NTI-chip did not impair the ability of tumor spheroids to secrete IL-8 **(Fig. S4cii)**.

**Supplementary Methods**

**Dextran Diffusion Assay in the NTI-chip**

To demonstrate that soluble factors can diffuse between different channels in the NTI-chip, we used 10kDa fluorescein-dextran (Invitrogen, D1821) whose molecular weight is comparable to that of human IL-8 (8.4 kDa), a neutrophil chemoattractant secreted by cancer cells, and that of human transforming growth factor-β (TGF-β) (12.5 kDa in monomers), a tumor-promoting cytokine secreted by neutrophils ^10–12^. First, 6 µL of empty collagen gel solution was loaded into both gel channels on ice and underwent gel polymerization at 37°C for 40 min. To study dextran diffusion from the tumor gel channel to the central channel, the upper medium channel was filled with 100 µL of 1 µM dextran solution in complete IMDM and the lower medium channel and the central channel were filled with 100 µL and 8 µL of complete IMDM respectively. To study dextran diffusion from the central channel to the tumor gel channel, the lower medium channel was filled with 100 µL of 1 µM dextran solution and the upper medium channel was filled with 100 µL of complete IMDM. The central channel was then filled with 8 µL of 1 µM dextran solution. Time-lapse imaging was performed on the Nikon ECLIPSE Ti2-E microscope at 37°C using brightfield and FITC channels at 10-min intervals for 12 h. Fluorescence intensity of the FITC images was measured along three random cross-sections of the tumor gel channel and the central channel at select time points in ImageJ and normalized by the maximum intensity values in the corresponding cross-sections.

**Testing fluid flow in the NTI-chip**

We used 10-µm green fluorescent particles (Thermo Scientific, 09-980-514) to test the presence of any unintentional fluid flow in the NTI-chip. First, 6 µL of empty collagen gel solution was loaded into both gel channels on ice and underwent gel polymerization at 37°C for 40 min. The two medium channels and the central channel were then filled with 100 µL and 8 µL of complete respectively. After 20-min stabilization, ~12,000 particles in 8 µL of complete IMDM were loaded into the central channel. Time-lapse imaging was performed on the Nikon ECLIPSE Ti2-E microscope using brightfield and FITC channels at 2.5-min intervals for 3 h. Automatic particle tracking was performed on FITC images using the TrackMate plugin in ImageJ to quantify the y-FMI and the mean velocity of particle movement as described in the section “Time-lapse Imaging and Analysis” in Methods. The baseline movement of the NTI-chip during time-lapse imaging was measured as the theoretical reference value by manual tracking using brightfield images in ImageJ.

**Transwell migration assay**

To assess the effect of CXCR2 antagonist AZD-5069 alone on dHL-60 cell migration in the absence of tumor spheroids, a transwell migration assay was performed using a transwell 24-well plate with polycarbonate membrane inserts of 3 µm pore size (Corning). The inserts and the bottom chambers were pre-coated with 2% bovine serum albumin (BSA, Sigma-Aldrich) for 1 hour at 37°C to prevent strong adhesion of dHL-60 cells. Coated inserts and wells were then rinsed with calcium- and magnesium-free Dulbecco's phosphate-buffered saline (DPBS, Gibco) twice to remove residual BSA. dHL-60 cells resuspended in serum-free IMDM at a density of 8x10^6^ cells/mL were pretreated with 1 µM AZD-5069 or with DMSO vehicle control on a rotator for 30 minutes at 37°C. dHL-60 cells were then seeded in 100 µl onto the inserts in the 24-well plate. Serum-free IMDM was gently added in 600 µl to the bottom chambers of the 24-well plate, avoiding any bubble formation between the insert and the liquid in the well. Cells were allowed to migrate for 4 hours at 37°C with 5% CO_2_. The numbers of dHL-60s that migrated into the bottom chambers were counted using an automated cell counter (﻿Bio-Rad) and the percentage of migrated dHL-60 cells was calculated.

**Immunofluorescence of Ki-67 in HL-60 cells and dHL-60 cells**

Immunofluorescence was performed to assess the expressions of proliferation marker Ki-67 by HL-60 cells before and after differentiation. HL-60 cells were differentiated into a neutrophil-like state (denoted “dHL-60 cells”) using 1.5% DMSO for 5 days. HL-60 cells and dHL-60 cells were seeded at a density of 10^6 cells/mL onto a glass-bottom 96-well plate (Cellvis) pre-coated with human fibronectin (﻿100 µg/mL in PBS) to promote cell attachment. After attachment for 1 h, cells were washed with PBS twice and fixed with 4% paraformaldehyde (Electron Microscopy Sciences) for 10 min at room temperature. The cells were then washed with PBS twice and permeabilized with 0.1% Triton X-100 for 20 min at room temperature. Cells were washed with PBS twice and blocked with 10% goat serum for 2 h at room temperature. The cells were then incubated overnight at 4°C with the mouse anti-Ki-67 (1:1000) primary antibody diluted in 10% goat serum. The cells were washed with PBS twice and incubated for 1 h at room temperature with the goat anti-mouse Alexa Fluor 488 secondary antibody (1:1000) and nuclei stain Hoechst 33342 (1:1000) diluted in 10% goat serum. Finally, the cells were washed with PBS twice and imaged with a Nikon ECLIPSE Ti2-E microscope ﻿using a Plan Apo 20X objective (NA = 0.80). Images were acquired using NIS-elements software and recorded using DAPI and FITC channels.

**Supplementary Tables**

| **Utility** | **Detail (cell-cell interactions or processes)** | **Reference** |
| --- | --- | --- |
| Investigation of the roles of soluble factors and direct contact in cell-cell interactions associated with tumor progression | Human pancreatic tumor spheroids and pancreatic stellate cells | [13] |
|  | Human colorectal tumor spheroids and cancer-associated fibroblasts (CAFs) | [14] |
|  | Human breast cancer cells and CAFs | [15] |
|  | Human liver cancer cells and bone marrow stromal cells | [16] |
|  | Human lung tumor spheroids and macrophages | [17] |
| Investigation of the role of neutrophils in processes associated with tumor progression | CAF modulation of neutrophil recruitment | [18] |
|  | Neutrophil extracellular trap (NET)-mediated ovarian tumor invasion | [19] |
|  | Formation of circulating tumor cell (CTC)-neutrophil clusters | [20] |
|  | Cancer cell extravasation | [21][22][23] |
|  | Brain metastasis of breast cancer | [24] |
|  | Bone metastasis of breast cancer | [25] |

**Table S1. Currently existing utilities of microphysiological systems to study tumor progression** ^13–17^ ^18–25^ .

**Supplementary Figures**

**
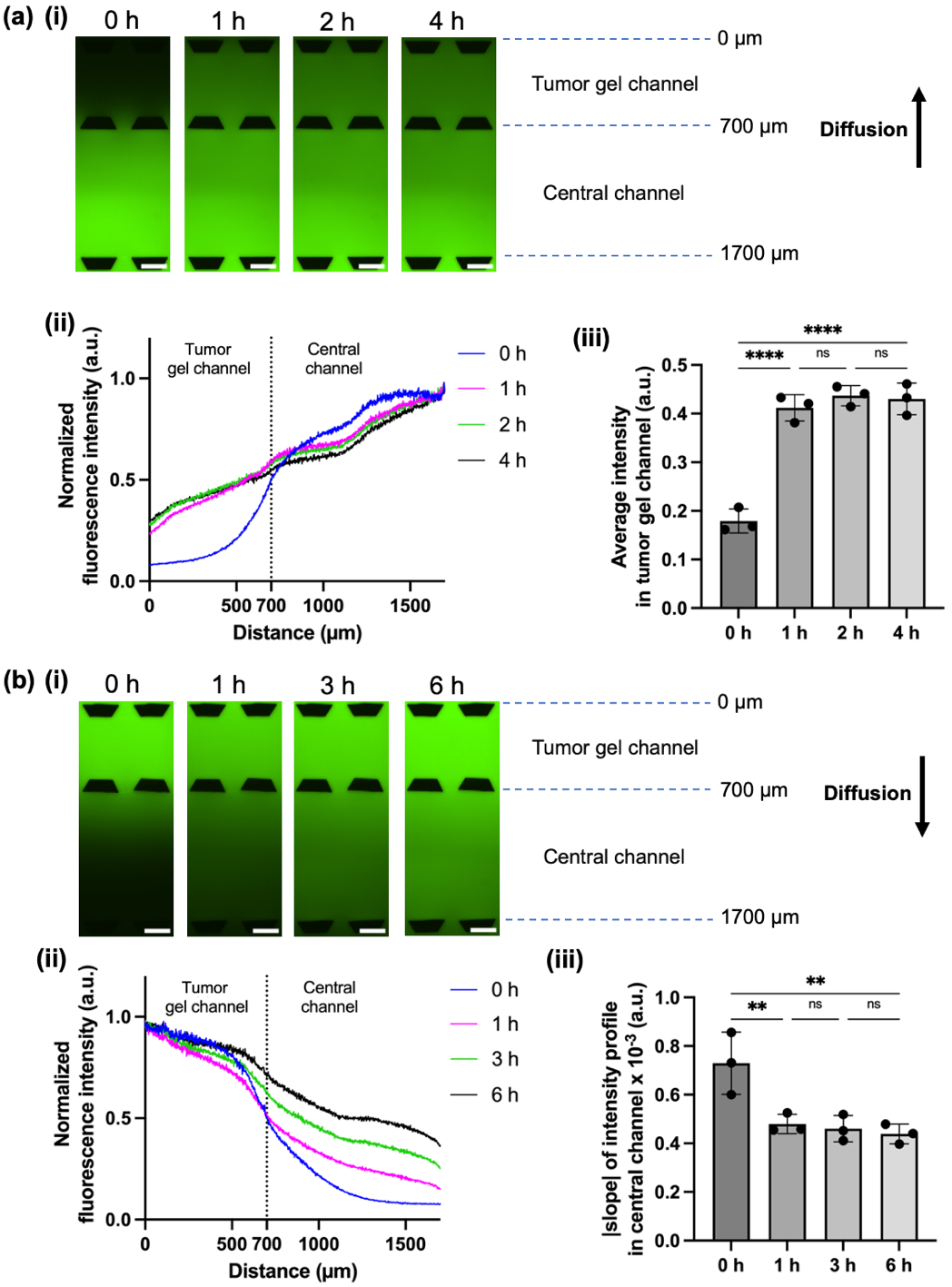
**

**Figure S1. Soluble factors can diffuse between the tumor gel channel and the central channel of the NTI-chip as demonstrated by 10kDa dextran. (a)** 10kDa fluorescein-dextran (1 µM) can diffuse from the central channel to the tumor gel channel within 1 h. **(i)** Representative fluorescent images showing distributions of dextran in the tumor gel channel and the central channel at t=0 h, 1 h, 2 h, and 4 h. scale bar, 200 µm. **(ii)** Normalized fluorescence intensity profiles along the cross-section of the tumor gel channel and the central channel at specified time points. **(iii)** Average normalized fluorescence intensity in the tumor gel channel at specified time points. The fluorescent intensity saturated after 1 h. Bars show mean ± SD. ****: p<0.0001, ns: p>0.05, one-way ANOVA with Tukey multiple comparisons test. **(b)** 10kDa fluorescein-dextran (1 µM) can diffuse from the tumor gel channel to the central channel and maintain a gradient in the central channel in the first 6 h. **(i)** Representative fluorescent images showing distributions of dextran in the tumor gel channel and the central channel at t=0 h, 1 h, 3 h, and 6 h. scale bar, 200 µm. **(ii)** Normalized fluorescence intensity profiles along the cross-section of the tumor gel channel and the central channel at specified time points. **(iii)** Absolute value of the slope of the normalized fluorescence intensity profile in the central channel (700-1200 µm) at specified time points. The slope remained constant from 1 h to 6 h. Bars show mean ± SD. **: p<0.01, ns: p>0.05, one-way ANOVA with Tukey multiple comparisons test. One experiment with three NTI-chips per condition was performed.


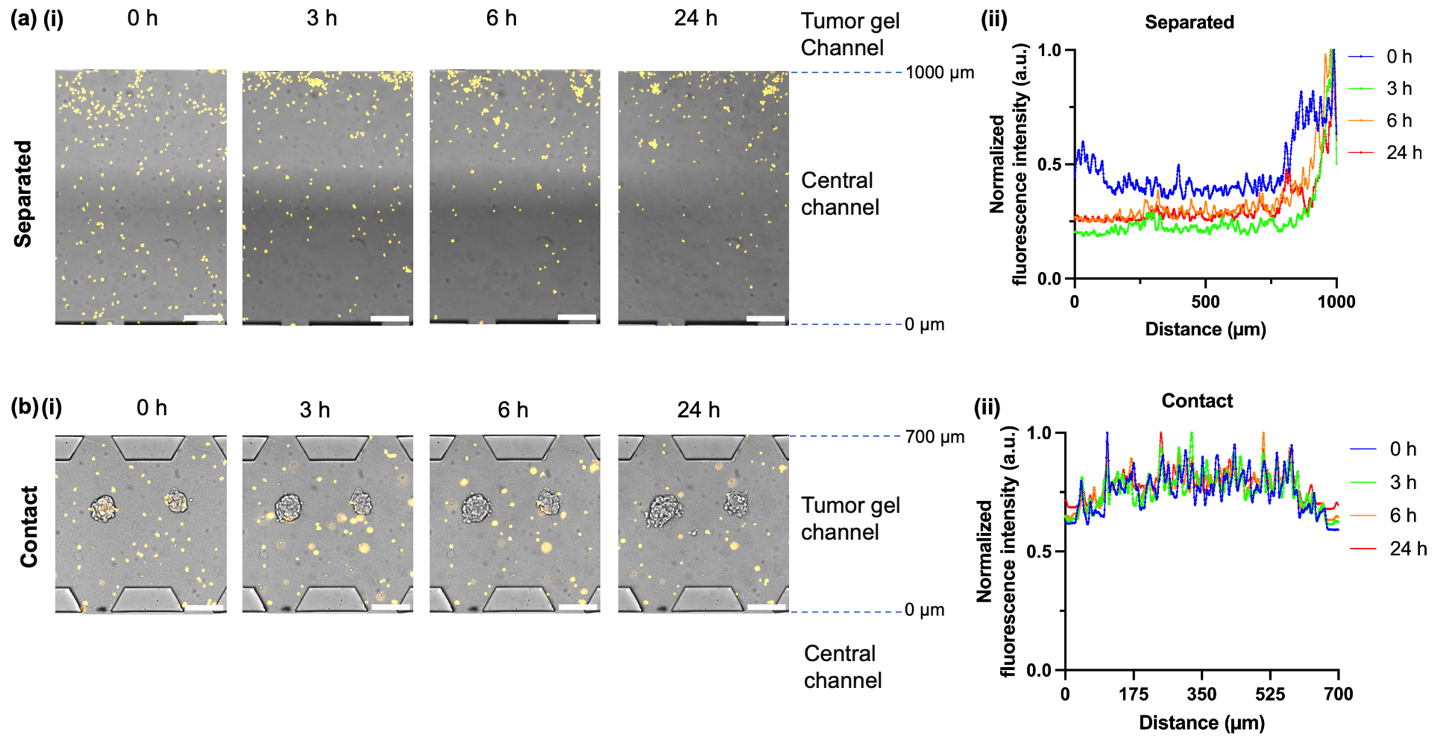


**Figure S2. Characterization of spatiotemporal distributions of neutrophils in the NTI-chip in both “separated” and “contact” scenarios. (a) (i)** Representative images showing positions of neutrophils (yellow) in the central channel in response to tumor spheroids (not shown) in the tumor gel channel at t=0 h, 3 h, 6 h, and 24 h of time-lapse imaging in the “separated” scenario. Scale bar, 150 µm. **(ii)** Normalized yellow fluorescence intensity profiles at select time points reflect that the spatial distribution of neutrophils across the central channel shifted upward towards the tumor gel channel over time. **(b) (i)** Representative images showing positions of neutrophils (yellow) and tumor spheroids (brightfield) in the tumor gel channel at t=0 h, 3 h, 6 h, and 24 h of time-lapse imaging in the “contact” scenario. Scale bar, 150 µm. **(ii)** Normalized yellow fluorescence intensity profiles at select time points reflect an overall symmetrical spatial distribution of neutrophils across the tumor gel channel over time. Hence, neutrophils demonstrated distinct behaviors between the two scenarios due to the different positioning of tumor spheroids in the NTI-chip. At leasts three independent experiments were performed and results from one representative experiment are shown.


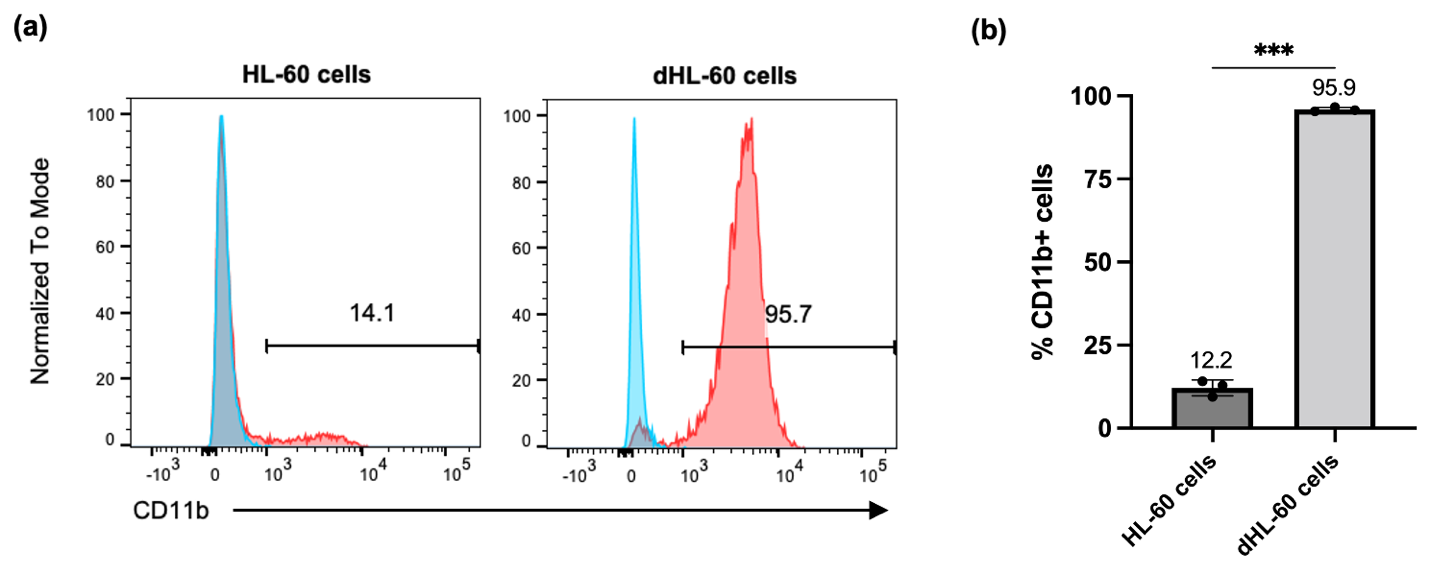


**Figure S3. HL-60 cells highly upregulate neutrophil surface marker CD11b after differentiation.** HL-60 cells were differentiated into a neutrophil-like state using 1.5% DMSO for 5 days. CD11b expressions by HL-60 cells (before differentiation) and dHL-60 cells (after differentiation) were measured by flow cytometry. **(a)** ﻿Histograms showing CD11b expression by HL-60 cells and dHL-60 cells from one representative experiment. Red histogram = stained sample; blue histogram = unstained control. The horizontal bars show percentages of CD11b-positive cells. **(b)** Bar plot showing the percentage of CD11b-positive cells in HL-60 cells and dHL-60 cells from three independent experiments. Bars show mean ± SD with mean values written above. ***: p<0.001, unpaired t test with Welch’s correction.


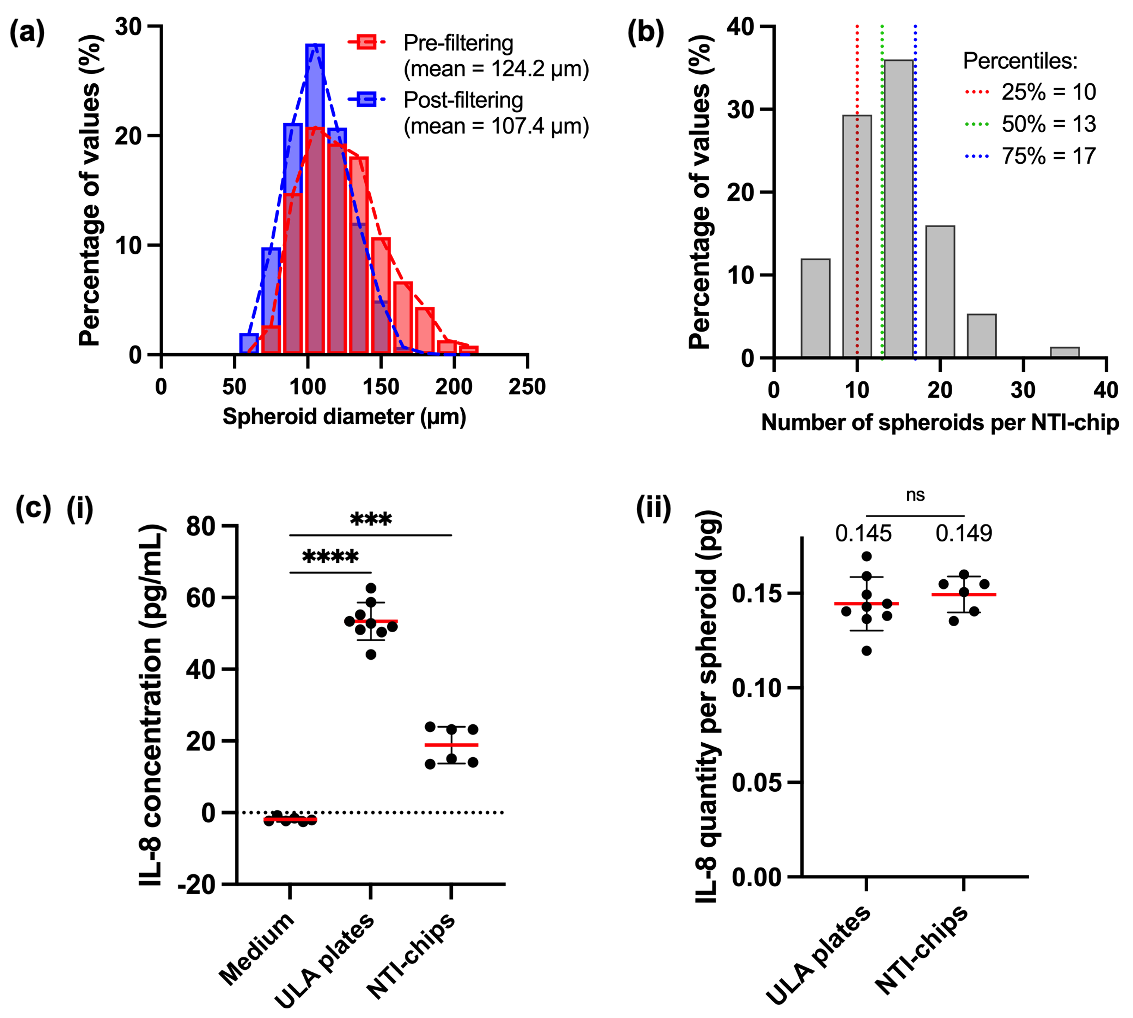


**Figure S4. Characterization and validation of tumor spheroid phenotypes in the NTI-chip. (a)** Frequency distributions of diameters of PANC-1 tumor spheroids on day 5 of culture before and after filtering through a 150 µm cell strainer. n= 549-596 spheroids per condition. **(b)** Frequency distribution of the number of tumor spheroids loaded into each NTI-chip in the “separated” scenario. NTI-chips with 10-17 spheroids were selected for analysis of neutrophil migration. **(c) (i)** IL-8 concentrations in the supernatants of PANC-1 tumor spheroids on ULA plates and in NTI-chips over 5 days and culture medium control were measured by ELISA. **(ii)** The quantity of IL-8 secreted per spheroid on ULA plates and in NTI-chips over 5 days was calculated. Data were collected from three independent experiments. Bars show mean ± SD with mean values shown above. ns: ≥ 0.05, ***: p<0.001, ****: p<0.0001, Brown-Forsythe and Welch ANOVA with Dunnett's T3 multiple comparisons test **(i)**; unpaired t test with Welch’s correction **(ii)**.

**
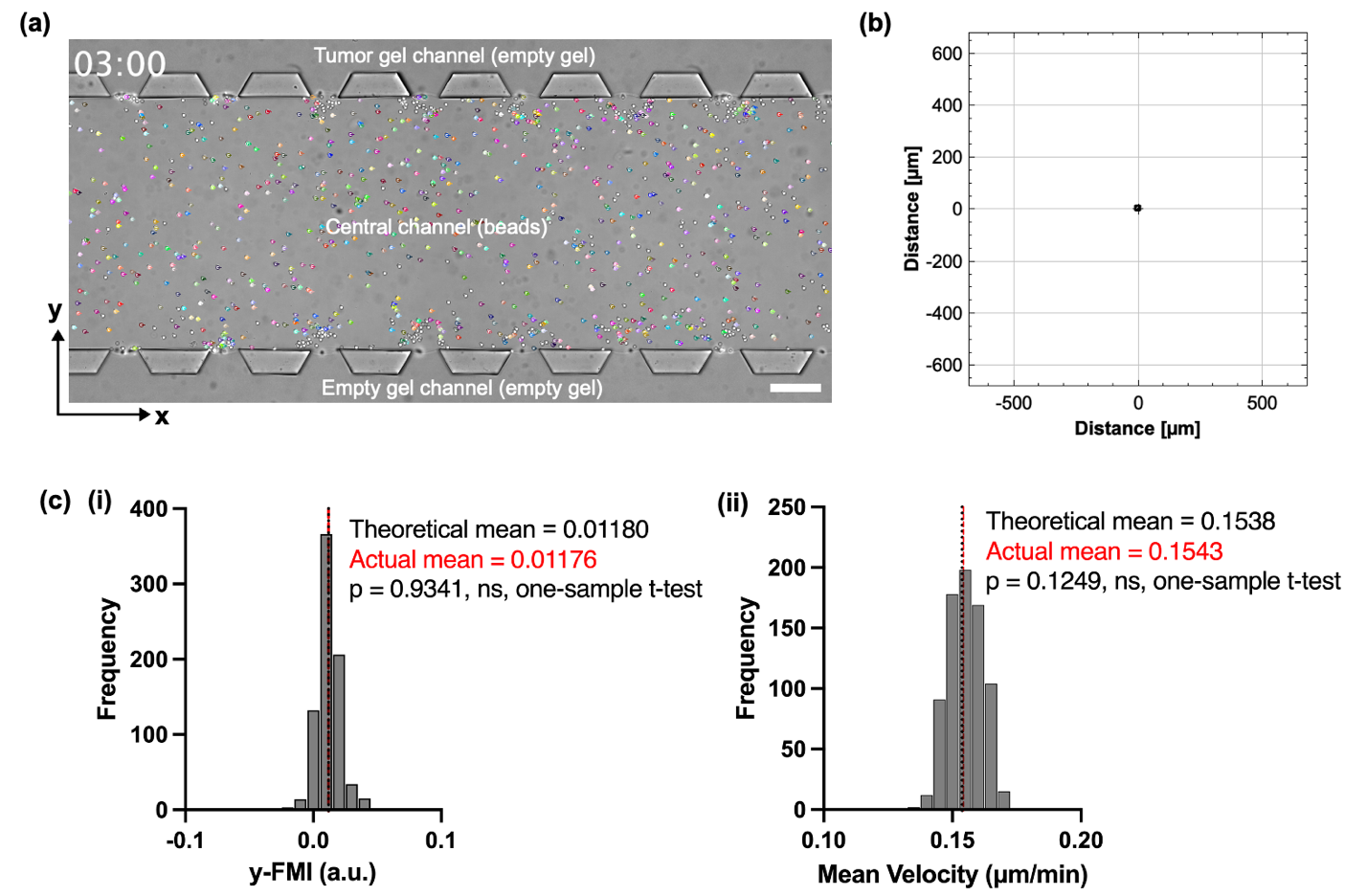
**

**Figure S5**. **Spontaneous fluid flow was not detected in the NTI-chip**. **(a)** A representative image showing 10-µm green fluorescent particles (pseudocolor-coded) in the central channel flanked by empty gel in both the tumor gel channel and the empty gel channel of the NTI-chip (brightfield), overlaid with single-bead trajectories over 3 h extracted by TrackMate (ImageJ). Fluorescent beads were loaded into the central channel 10 min after the NTI-chip was filled with culture medium. Scale bar, 200 µm. **(b)** A representative trajectory plot of fluorsecent beads in the NTI-chip over 3 h. 778 beads were tracked. The trajectories are clustered at the origin due to the beads being overall stationary/immobile. **(c)** Frequency distributions of the y-FMI **(i)** and mean velocity **(ii)** of fluorescent beads. The theoretical mean was measured by manually tracking the baseline movement of the NTI-chip in the background (brightfield) during time-lapse imaging. The theoretical mean is shown at the black vertical line and the actual mean of the sample at the red vertical line. ns: p≥ 0.05, one-sample t-test. The actual mean was not statistically different from the theoretical mean, suggesting that the beads did not move relative to the chip itself and thus disproving the existence of any unintentional fluid flow. Two independent experiments were performed and results from one representative experiment are shown.


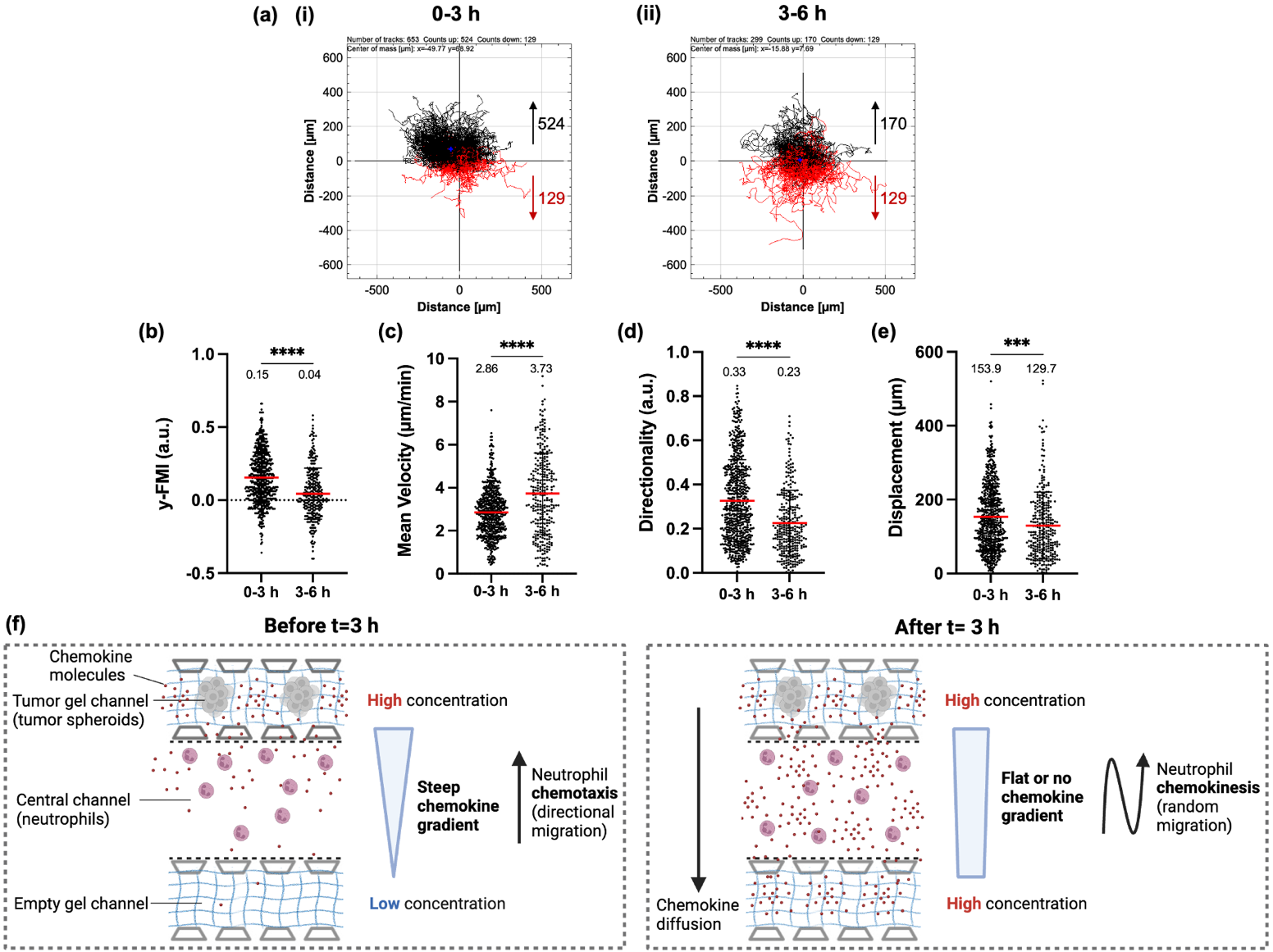


**Figure S6. dHL-60 cell migration in response to PANC-1 tumor spheroids switches from chemotaxis to chemokinesis after 3 h in the “separated” scenario.** **(a)** Representative trajectory plots of migrating dHL-60 cells treated with DMSO vehicle control in response to PANC-1 tumor spheroids during 0-3 h **(i)** and 3-6 h **(ii)**. Numbers in black and in red represent numbers of dHL-60 cells migrating toward and away from tumor spheroids, respectively. **(b-e)** Scatter plots depicting the y-FMI **(b)**, mean velocity **(c)**, directionality **(d)**, and displacement **(e)** of migrating dHL-60 cells during 0-3 h and 3-6 h. Each data point represents a single cell and n= 299-653 cells tracked per condition. Bars show mean (red) ± SD (black) with mean values written above the points. At least three independent experiments were performed and results from one representative experiment are shown. ***: p<0.001, ****: p<0.0001, unpaired t test with Welch’s correction. **(f)** Schematic showing continual diffusion of tumor-secreted chemokines and flattening of the chemokine gradient as a possible explanation for the switch in dHL-60 cell migration mode from chemotaxis to chemokinesis. Created with BioRender.com.


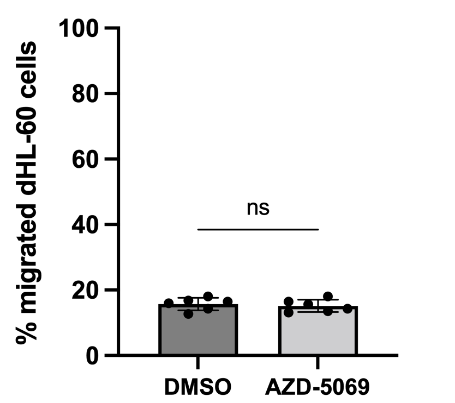


**Figure S7. CXCR2 inhibition via AZD-5069 alone does not affect dHL-60 cell migration in a transwell assay.** dHL-60 cells pre-treated with DMSO vehicle control or 1 μM AZD-5069 were allowed to migrate into the bottom chambers of the transwells containing serum-free culture medium for 4 hours. The bar plot shows the percentage of dHL-60 cells that migrated into bottom chambers in DSMO and AZD-5069 conditions. Bars show mean ± SD of n= 6 wells per condition in one experiment. ns: ≥ 0.05, unpaired t test with Welch’s correction.


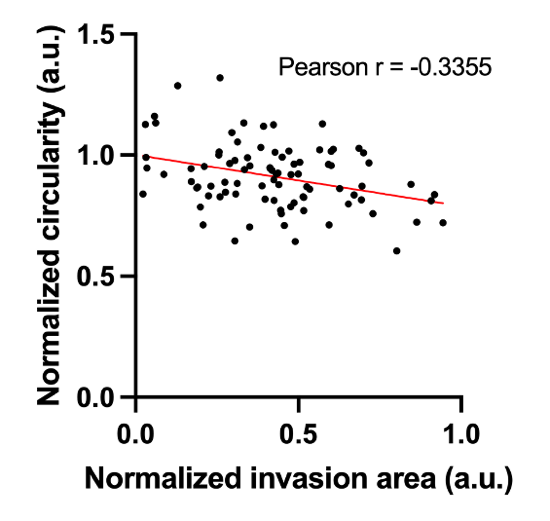


**Figure S8. Significant negative correlation between normalized circularity and normalized invasion area of PANC-1 tumor spheroids undergoing spontaneous invasion in the NTI-chip.** Scatter plot showing the normalized circularity and the normalized invasion area of tumor spheroids that invaded in the collagen gel matrix in the absence of AZD-5069 and dHL-60 cells. n=88 spheroids. The red line shows the simple linear regression line. At least three independent experiments were performed. p<0.01 for Pearson correlation coefficient r = -0.3355, meaning that the correlation is statistically significant.


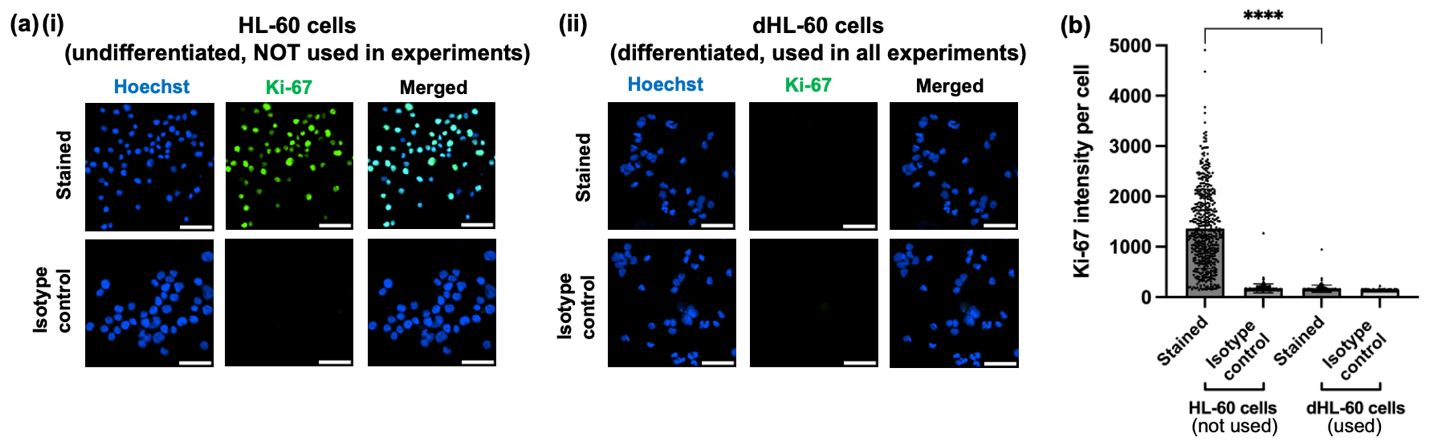


**Figure S9. HL-60 cells do not express proliferation marker Ki-67 after differentiation.** HL-60 cells were differentiated into a neutrophil-like state using 1.5% DMSO for 5 days before interacting with tumor spheroids in the NTI-chip. Ki-67 expressions by HL-60 cells (before differentiation) and dHL-60 cells (after differentiation) were examined by immunofluorescence. **(a)** Representative 20X fluorescence images of HL-60 cells and dHL-60 cells (blue, Hoechst) immunostained for Ki-67 (green). Scale bar, 50 µm. Samples stained with the secondary antibody only were used as isotype control. **(b)** A bar plot showing the Ki-67 fluorescence intensity per cell in specified conditions. Each data point represents a cell and n = 170–465 cells per condition. Bars show mean ± SD from one experiment. ****: p<0.0001, unpaired t test with Welch’s correction. Ki-67 intensity per cell was significantly reduced in dHL-60 cells compared to HL-60 cells.


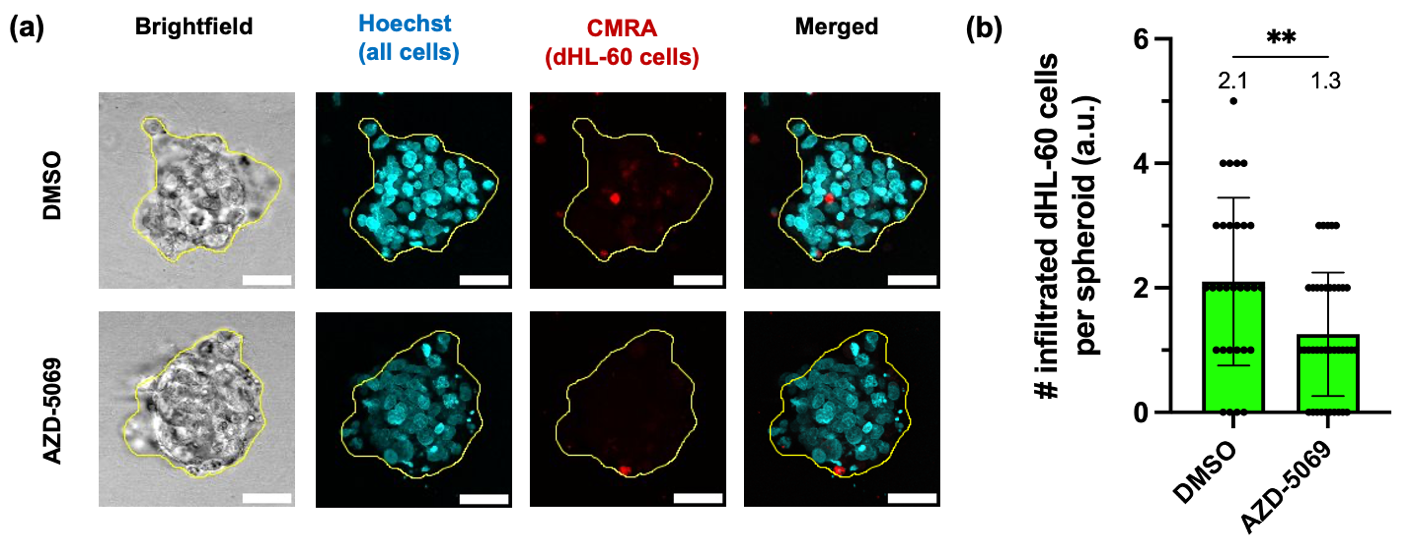


**Figure S10. CXCR2 inhibition reduces the infiltration of dHL-60 cells in PANC-1 tumor spheroids at t= 24 h in the “contact” scenario. (a)** Representative confocal images showing the infiltration of dHL-60 cells (red, CMRA; blue, Hoechst) treated with DMSO vehicle control or 1 μM AZD-5069 in PANC-1 tumor spheroids (blue, Hoechst) fixed at t=24 h in the “contact” scenario. The boundary of tumor spheroids (yellow) was manually defined using brightfield images. Any dHL-60 cells that entered the boundary are considered to have infiltrated in the spheroid. Fluorescence images are maximum intensity projections of z-stacks with a 2 μm step size. Scale bar, 50 µm. **(b)** A bar plot showing the number of infiltrated dHL-60 cells per tumor spheroid at t= 24 h in specified conditions. Each data point represents a spheroid and n = 30-39 spheroids per condition. Bars show mean ± SD with mean values written above the points. **: p<0.01, unpaired t test with Welch’s correction.

**Supplementary Video Legends**

**Supplementary Video 1**. Representative video showing dHL-60 cells (yellow, CMRA dye) treated with DMSO vehicle control interacting with PANC-1 tumor spheroids (brightfield) during 12 h of time-lapse imaging in the “contact” scenario in the NTI-chip. The video is overlaid with dHL-60 cell tracks extracted by TrackMate (ImageJ). Any dHL-60 cells that entered the circular ROI (yellow) around the spheroid are considered to be in contact with the spheroid. For the particular tumor spheroid shown in this video, the frequency of contact with dHL-60 cells is 43 and the mean duration of contact is 41.8 min. Scale bar, 50 µm.

**Supplementary Video 2**. Representative video showing dHL-60 cells (yellow, CMRA dye) treated with AZD-5069 (1 µM) interacting with PANC-1 tumor spheroids (brightfield) during 12 h of time-lapse imaging in the “contact” scenario in the NTI-chip. The video is overlaid with dHL-60 cell tracks extracted by TrackMate (ImageJ). Any dHL-60 cells that entered the circular ROI (yellow) around the spheroid are considered to be in contact with the spheroid. For the particular tumor spheroid shown in this video, the frequency of contact with dHL-60 cells is 21 and the mean duration of contact is 13.5 min. Scale bar, 50 µm.

**References:**

1. Blair, O. C., Carbone, R. & Sartorelli, A. C. Differentiation of HL-60 promyelocytic leukemia cells: Simultaneous determination of phagocytic activity and cell cycle distribution by flow cytometry. *Cytometry* **7**, 171–177 (1986).

2. Jung, Y.-J. *et al.* Investigation of Chemotactic Activities in Differentiated HL-60 Cells by a Time-lapse Videomicroscopic Assay. *Immune Netw* **6**, 76 (2006).

3. Collins, S. J., Ruscetti, F. W., Gallagher, R. E. & Gallo, R. C. Terminal differentiation of human promyelocytic leukemia cells induced by dimethyl sulfoxide and other polar compounds. *Proc Natl Acad Sci U S A* **75**, 2458–2462 (1978).

4. Rincón, E., Rocha-Gregg, B. L. & Collins, S. R. A map of gene expression in neutrophil-like cell lines. *BMC Genomics* **19**, (2018).

5. Guo, Y. *et al.* Differentiation of HL‑60 cells in serum‑free hematopoietic cell media enhances the production of neutrophil extracellular traps. *Exp Ther Med* **21**, (2021).

6. Baggiolini, M. & Clark-Lewis, I. Interleukin-8, a chemotactic and inflammatory cytokine. *FEBS Lett* **307**, 97–101 (1992).

7. Jones, C. N. *et al.* Microfluidic chambers for monitoring leukocyte trafficking and humanized nano-proresolving medicines interactions. *Proc Natl Acad Sci U S A* **109**, 20560–20565 (2012).

8. Teijeira, A. *et al.* IL8, neutrophils, and NETs in a collusion against cancer immunity and immunotherapy. *Clinical Cancer Research* vol. 27 2383–2393 Preprint at https://doi.org/10.1158/1078-0432.CCR-20-1319 (2021).

9. Teijeira, Á. *et al.* CXCR1 and CXCR2 Chemokine Receptor Agonists Produced by Tumors Induce Neutrophil Extracellular Traps that Interfere with Immune Cytotoxicity. *Immunity* **52**, 856-871.e8 (2020).

10. Aoyagi, Y. *et al.* Overexpression of TGF-β by infiltrated granulocytes correlates with the expression of collagen mRNA in pancreatic cancer. *British Journal of Cancer 2004 91:7* **91**, 1316–1326 (2004).

11. Ungefroren, H. *et al.* The Quasimesenchymal Pancreatic Ductal Epithelial Cell Line PANC‐1—A Useful Model to Study Clonal Heterogeneity and EMT Subtype Shifting. *Cancers (Basel)* **14**, 2057 (2022).

12. Wang, H. *et al.* Transforming growth factor β-induced epithelial-mesenchymal transition increases cancer stem-like cells in the PANC-1 cell line. *Oncol Lett* **3**, 229 (2012).

13. Lee, J. H. *et al.* Microfluidic co-culture of pancreatic tumor spheroids with stellate cells as a novel 3D model for investigation of stroma-mediated cell motility and drug resistance. *Journal of Experimental and Clinical Cancer Research* **37**, (2018).

14. Jeong, S. Y., Lee, J. H., Shin, Y., Chung, S. & Kuh, H. J. Co-culture of tumor spheroids and fibroblasts in a collagen matrix-incorporated microfluidic chip mimics reciprocal activation in solid tumor microenvironment. *PLoS One* **11**, (2016).

15. Truong, D. *et al.* Breast cancer cell invasion into a three dimensional tumor-stroma microenvironment. *Sci Rep* **6**, 1–18 (2016).

16. Menon, N. V., Chuah, Y. J., Cao, B., Lim, M. & Kang, Y. A microfluidic co-culture system to monitor tumor-stromal interactions on a chip. *Biomicrofluidics* **8**, (2014).

17. Bai, J. *et al.* Contact-dependent carcinoma aggregate dispersion by M2a macrophages via ICAM-1 and ß2 integrin interactions. *Oncotarget* **6**, 25295–25307 (2015).

18. Yu, J. *et al.* A reconfigurable microscale assay enables insights into cancer-associated fibroblast modulation of immune cell recruitment. *Integrative Biology* **13**, 87–97 (2021).

19. Surendran, V., Rutledge, D., Colmon, R. & Chandrasekaran, A. A novel tumor-immune microenvironment (TIME)-on-Chip mimics three dimensional neutrophil-tumor dynamics and neutrophil extracellular traps (NETs)-mediated collective tumor invasion. *Biofabrication* **13**, (2021).

20. Park, J., Park, S., Hyun, K. A. & Jung, H.-I. Microfluidic recapitulation of circulating tumor cell–neutrophil clusters via double spiral channel-induced deterministic encapsulation. *Lab Chip* (2021) doi:10.1039/d1lc00433f.

21. Chen, M. B. *et al.* Inflamed neutrophils sequestered at entrapped tumor cells via chemotactic confinement promote tumor cell extravasation. *Proc Natl Acad Sci U S A* **115**, 7022–7027 (2018).

22. Spiegel, A. *et al.* Neutrophils suppress intraluminal NK cell-mediated tumor cell clearance and enhance extravasation of disseminated carcinoma cells. *Cancer Discov* **6**, 630–649 (2016).

23. Crippa, M. *et al.* A microfluidic model of human vascularized breast cancer metastasis to bone for the study of neutrophil-cancer cell interactions. *Mater Today Bio* **17**, (2022).

24. Crippa, M. *et al.* A microphysiological early metastatic niche on a chip reveals how heterotypic cell interactions and inhibition of integrin subunit β3 impact breast cancer cell extravasation. *Lab Chip* **21**, 1061–1072 (2021).

25. Safarulla, S., Madan, A., Xing, F. & Chandrasekaran, A. CXCR2 Mediates Distinct Neutrophil Behavior in Brain Metastatic Breast Tumor. *Cancers (Basel)* **14**, (2022).
